# Du’an Karst of Guangxi: a kingdom of the cavernicolous genus *Dongodytes* Deuve (Coleoptera, Carabidae, Trechinae)

**DOI:** 10.3897/zookeys.454.7269

**Published:** 2014-11-14

**Authors:** Mingyi Tian, Haomin Yin, Sunbin Huang

**Affiliations:** 1 Department of Entomology, College of Natural Resources and Environment, South China Agricultural University, 483 Wushan Road, Guangzhou, 510642, China

**Keywords:** Ground beetles, anophthalmic, troglobitic, new species, specific diversity, China

## Abstract

Recent cave biodiversity surveys carried out in Du’an County and its adjacent areas of northwestern Guangxi, China, have revealed some exciting scientific findings. In a very limited area seven new species of the cavernicolous trechine genus *Dongodytes* Deuve, 1993 were found and are described: Dongodytes (s. str.) elongatus **sp. n.**, Dongodytes (s. str.) troglodytes **sp. n.**, Dongodytes (s. str.) lani **sp. n.**, Dongodytes (Dongodytodes) brevipenis **sp. n.**, Dongodytes (Dongodytodes) jinzhuensis **sp. n.**, Dongodytes (Dongodytodes) inexpectatus **sp. n.** and Dongodytes (Dongodytodes) yaophilus **sp. n.** Diagnoses and notes on the genus, subgenera, and two known species in Du’an Karst, Dongodytes (s. str.) baxian Tian, 2011 and Dongodytes (Dongodytodes) deharvengi Tian, 2011, are also given. A key to subgenera and all species of *Dongodytes* is provided. To date, *Dongodytes* becomes one of the richest in species genera of subterranean carabid trechines in China with 12 species which are arranged into two subgenera. *Dongodytes* (s. str.) Deuve is composed of seven species, four of which from Du’an County, each of other three from Bama, Fengshan and Tian’e Counties, respectively. All species of the subgenus *Dongodytodes* Tian, 2011 are recorded from Du’an Karst. By having 10 species (nine *Dongodytes* and one *Libotrechus* Uéno, 1998), Du’an Karst holds the richest specific diversity of cavernicolous Trechinae in China. *Dongodytes* species are distributed in a very limited area of the river Hongshui He drainages in northwestern Guangxi, and the river acts as a natural barrier of *Dongodytes* dispersal at only a specific level. However, all members of *Dongodytodes* are recorded from the eastern or northern bank of Hongshui He.

## Introduction

Although troglobitic trechine species were not reported from Mainland China before 1991 (Uéno and Wang 1991), China has become one of the most fascinating countries for subterranean trechines in the world, having more than 30 genera and about 90 species so far (Tian and Clarke 2012). All of the known cave-dwelling trechines in Mainland China are recorded from the southern provinces or regions, viz. Guizhou, Guangxi, Yunnan, Sichuan, Chongqing, Hunan, Hubei, Jiangxi and Zhejiang (Deuve et al. 1999; Deuve 2002; Uéno 1998b, 2007; Uéno and Clarke 2007; Deuve and Tian 2008, 2009, 2011; Tian 2010, 2011; Tian and Yin 2013).

*Dongodytes* Deuve, 1993 is one of the morphologically most modified cavernicolous genera within the subfamily Trechinae and represents a very peculiar lineage (Deuve 1993; Vigna Taglianti 1997; Uéno 1998a). It was established by Deuve (1993) to comprise the species *Dongodytes fowleri* Deuve, 1993, known by only a single male specimen at that time which was collected in a limestone cave in Bama County of northwestern Guangxi. Then, Uéno (1998a) discovered two more specimens of *Dongodytes fowleri* in the same cave and described the second species *Dongodytes grandis* from a cave in Fengshan County. Seven years later, Uéno (2005) reported the third species, *Dongodytes giraffa* from a cave in southern Tian’e County, northern Guangxi, which is the most modified species in *Dongodytes*, having a very long and elongate head and prothorax, and serrate humeral shoulders. According to material collected from two caves in Du’an County, Tian (2011) added two species to the genus, one belonging to the nominate subgenus *Dongodytes* (s. str.), the other to the newly established subgenus *Dongodytodes* Tian, 2011.

**Figure 1.**
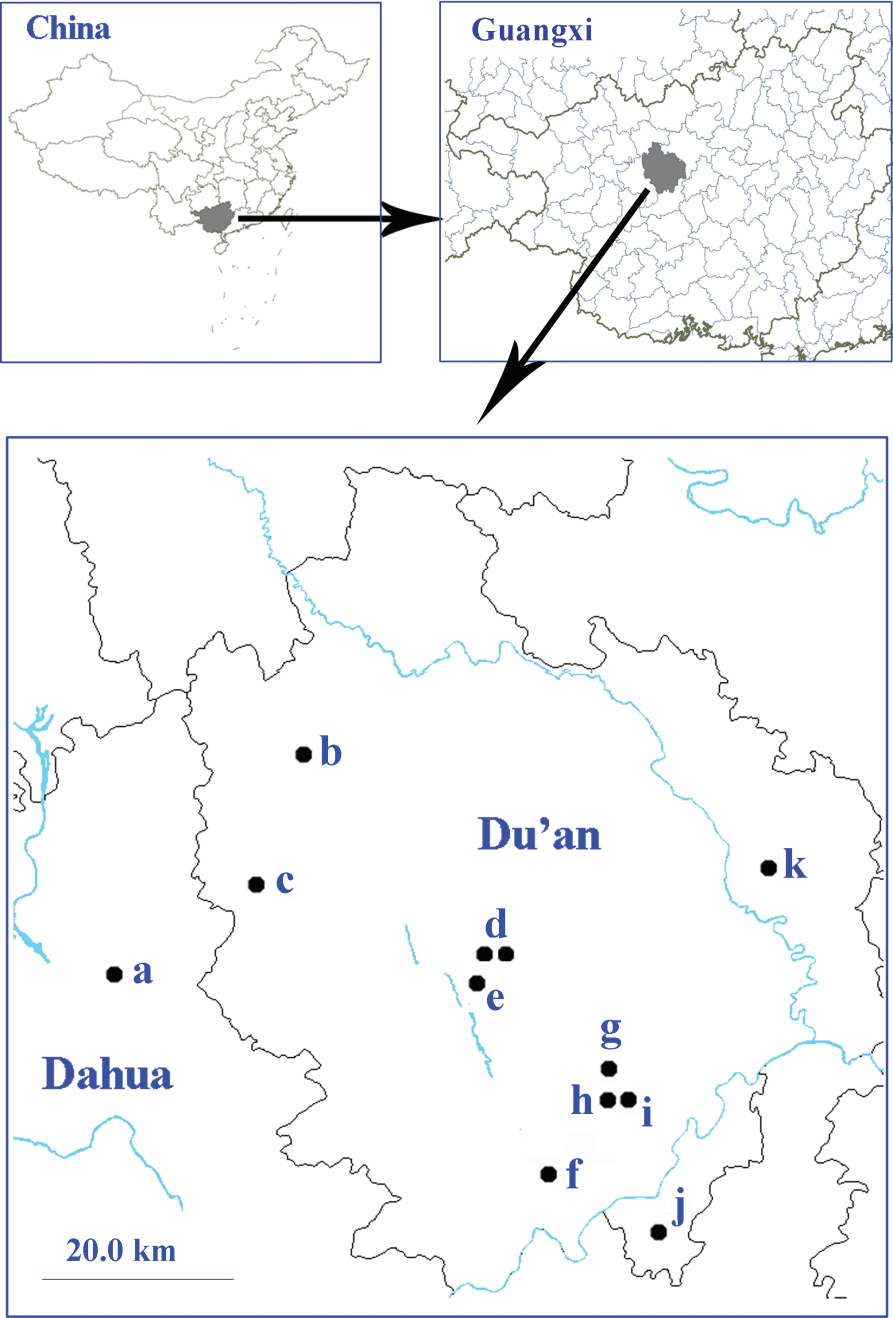
Surveyed caves in Du’an Karst from which trechine beetles were discovered. **a** Qiaoxu Dong (Qibainong) **b** Lubian Dong (Xia’ao) **c** Shuiyuan Dong (Longfu) **d** Jinzhu Dong I and Jinzhu Dong II **e** Nongguanshang Dong II **f** Baxian Dong (Chengjiang) **g** Diaomao Dong (Chengjiang) **h** Nongzhong Dong I (Chengjiang) **i** Nongqu Dong (Longwan) **j** Longhuan Dong (Longwan) **k** Lapo Dong I (Lalie).

**Figures 2–5.**
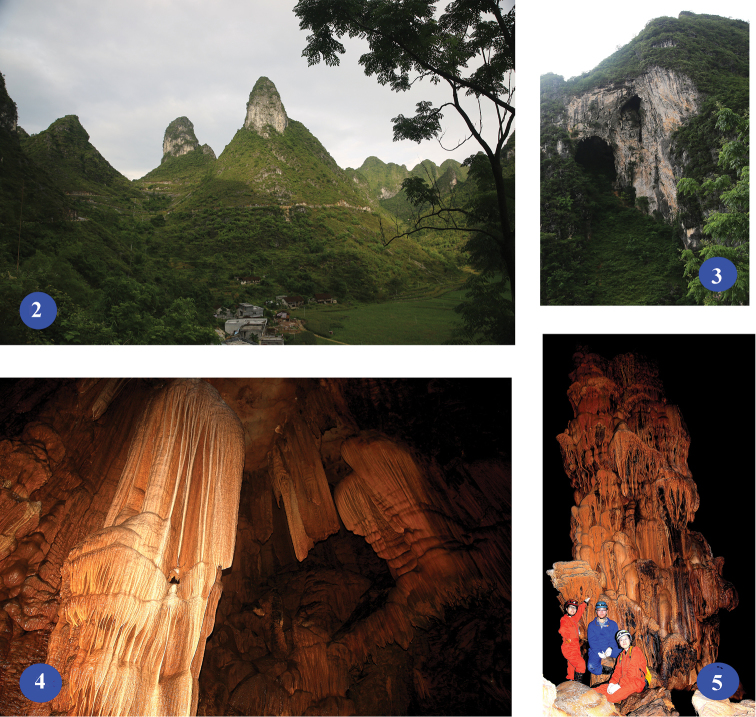
Karstic landscapes in Du’an County. **2** hills near Gushan, on the road to Lalie **3** a cave entrance near Gushan, on the road to Lalie **4–5** beautiful deposits in Qinqi Dong of Longwan.

Administratively, Du’an Yao Autonomous County belongs to Hechi Prefecture, northwestern Guangxi Zhuang Autonomous Region (Fig. 1). This county is located on the transition zones between Yunnan-Guizhou Plateau and Guangxi Basin. Karstic landscape covers 77.9% of the whole terrestrial areas in Du’an (Hu et al. 2012), creating numerous mountains, hills and caves (Figs 2–5). However, the subterranean fauna in Du’an is still not well-known. The only exception is the fauna of cave fishes. In total, nine species of cave fishes are recorded in Du’an, including three anophthalmic species (Lan et al. 2013). On the contrary, only three troglobitic carabid beetles have been reported from this county so far: a clivinine *Guiodytes cavicola* Tian, 2013 (Tian 2013, 2014) and two trechines, Dongodytes (s. str.) baxian Tian, 2011 and Dongodytes (Dongodytodes) deharvengi Tian, 2011. All above mentioned ground beetles were part of the findings of the China-France Biospeleological Expedition 2010 in Guangxi, which was led by Dr. Louis Deharveng, a well known biospeleologist of Muséum National d’Histoire Naturelle, Paris, and organized by the Biodiversity Conservation Office, Forestry Department of Guangxi Regional Government and financed by the World Bank, GEF.

In 2013, Du’an Karst was surveyed to study the cave fauna as part of a biodiversity conservation project, sponsored mainly by Nanjing Institute of Environmental Sciences, Ministry of Environmental Protection. Three biospeleological surveys had been carried out in May, June and December of 2013 in Du’an and the adjacent karstic areas, and led to important discoveries. Seven new species of *Dongodytes* were found: three belonging to *Dongodytes* (s. str.), and four to the subgenus *Dongodytodes*. In addition, another new species of the genus *Libotrechus* Uéno, 1998 was also found in cave Shuiyuan Dong in Longfu (Lin and Tian 2014). This extremely rich anophthalmic trechine fauna, of 10 species, makes Du’an Karst the “hottest” spot of cavernicolous trechines in China. It is probably that additional *Dongodytes* species or other eyeless trechines will be discovered in Du’an Karst.

## Materials and methods

During the biospeleological surveys in 2013, we visited and collected in 48 caves in Du’an and its adjacent areas. In total, 95 specimens of *Dongodytes* were found in 12 caves, all of them are in Du’an County except the cave Qiaoxu Dong which is in Dahua County but belongs also to Du’an Karst (Fig. 1a).

The beetles were collected by hand or by using an aspirator, and kept in 55% ethanol before study. Dissections and observations were made under a Leica MZ75 dissecting microscope. Dissected genital pieces, including the median lobe and parameres of aedeagus, were glued on small paper cards and then pinned under the specimen from which they were removed. Digital pictures were taken using a Canon EOS 5D Mark III camera, and then processed by means of Adobe Photoshop CS5 software. Distributional map was prepared using Mapinfo 8.5 SCP software.

Length of body is measured from apex of right mandible (in opened position) to elytral apex.

Abbreviations of other measurements used in the text are as following.

HL length of head, from apex of right mandible to occipital suture;

HW maximum width of head;

PL length of pronotum, along the median line;

PW maximum width of pronotum;

PTW maximum width of prothorax;

PAW width of pronotum at front;

PBW width of pronotum at base;

EL length of elytra, from base of scutellum to elytral apex;

EW maximum width of combined elytra.

Abbreviations for the specimens’ depository are as following.

IOZ National Museum of Zoology, Institute of Zoology, Chinese Academy of Sciences, Beijing;

MNHN Muséum National d’Histoire Naturelle, Paris;

SCAU South China Agricultural University, Guangzhou;

ZUBM Biological Museum of Zhongshan University, Guangzhou.

## Systematics

### Genus Dongodytes

Taxon classificationAnimaliaColeopteraCarabidae

Deuve

Dongodytes Deuve, 1993: 292

#### Type species.

*Dongodytes fowleri* Deuve, 1993: 292

#### Type locality.

Jiabao Dong, in Bama County, northwestern Guangxi (Uéno 1998).

#### Diagnostic characters of the genus.

Members of *Dongodytes* share the following combined characters: elytra remarkably elongate though much wider than prothorax and without shoulders; eyes completely effaced and depigmented; body especially head and prothorax strongly elongate; frontal furrows very short, mandibles long and slender, right mandible bidentate, palps and antennae very elongate and thin; propleura strongly tumid, visible from above; pronotum much longer than wide; elytra depressed medially on humeral parts, anterior and preapical dorsal pores present or not, humeral set of marginal umbilicate pores not aggregated, 1^st^ pore widely located from 2^nd^ and 3^rd^ which are close to each other, 1^st^ to 3^rd^ more or less adjoining marginal gutter, 4^th^ distinctly dorsal wards located and far from marginal gutter; 5^th^ and 6^th^ of middle set are close to each other; legs thin and very long, tarsi slender; protarsi not distinctly modified in male.

Male genitalia are strongly diversified in *Dongodytes* and could be important character states for phylogenetic analysis of the genus. Three types of the median lobe can be recognized. Type I, species of the subgenus *Dongodytes* (s. str.) which are known only from Du’an Karst, the median lobe is very short and stout, basal orifice very large, but with a very small, sometimes indistinct, sagittal aileron, and the parameres are broader (Figs 33–38); Type II, species of the subgenus *Dongodytodes*, which are all known from Du’an Karst, the median lobe is very elongate and thin, basal orifice comparatively small, but with a large sagittal aileron, and slender parameres (Figs 39–46); Type III, species of *Dongodytes* (s. str.) which are all known from other counties rather than Du’an: the median lobe is intermediately shaped between the two patterns described above, moderately elongate, rather stout, and with a large sagittal aileron; in particular, the median lobe distinctly curved at apex (Deuve 1993: Fig. 2; Uéno 1998: Figs 1–4).

#### Systematic position of *Dongodytes*.

*Dongodytes* is one of the most modified troglobiomorphic genera of trechines in the world. To determine the taxonomic position of *Dongodytes* within the tribe Trechini remains a challenge. Vigna Taglianti (1997) and Uéno (1998) suggested it may be allied to the European *Aphaenops* series, contrary to Deuve (1993) who compared *Dongodytes* with *Sinaphaenops* Uéno & Wang, 1991. Because recent study based on molecular phylogenetic analysis have clearly demonstrated that the *Aphaenops* series in the widest and traditional sense are restricted to the Pyrenean lineage (Faille et al. 2010, 2013), we agree with Deuve’s opinion. *Dongodytes* seems to be allied to its Chinese congeners such as *Sinaphaenops* and *Pilosaphaenops* Deuve & Tian, 2008. For example, by having very short frontal furrows, very elongate genae and slender neck constriction, the head structures of *Dongodytes* are more or less similar to those of *Sinaphaenops* and *Pilosaphaenops*. Furthermore, all of the above genera have similar prothorax although latero-marginal setae are always absent in *Sinaphaenops* and *Pilosaphaenops*.

#### Geographical distribution.

Endemic to northwestern Guangxi (Fig. 73). Members of *Dongodytes* are recorded from several counties of Hechi Prefecture. However, only a single species is known in each of Bama, Fengshan, Tian’e and Dahua Counties, respectively. On the other hand, majority of the species (eight) are distributed in Du’an County. Thus, it is clear that, from the present knowledge, all species of *Dongodytes* are distributed in a very limited area of the river Hongshui He drainages in northwestern Guangxi. The river acts as a natural barrier for dispersal of *Dongodytes* at only a specific level. The nominate subgenus *Dongodytes* (s. str.) covers a larger distribution range than the subgenus *Dongodytodes* which is restricted to Du’an Karst in the eastern or northern bank of Hongshui He.

#### Taxonomic treatment.

Species of *Dongodytes* are divided into two subgenera, *Dongodytes* (s. str.) Deuve and *Dongodytodes* Tian. Both subgenera can be separated each other by shape of head, length of antennae, body pubescent or not, and chaetotaxal pattern on head, pronotum, elytra, and abdominal ventrite VII of male (Tian 2011). See the following key for details.

#### Key to subgenera and species of *Dongodytes* Deuve

**Table:** 

1	Antennae very long, extending over elytral apex (Figs 8–10), head more elongate, gradually narrowed posteriad, neck constriction long (Figs 16–19), posterior supraorbital setae present or not, head and pronotum glabrous or covered with sparse long and erected setae; elytra glabrous in most species (except for *Dongodytes elongatus* sp. n.), anterior dorsal pore of 3^rd^ elytral stria present (Fig. 6); ventrite VII in male with two pairs of paramedian setae (Fig. 25) (subgenus *Dongodytes* Deuve)	**2**
–	Antennae short, not extending over elytral apex (Figs 11–15), head less elongate, suddenly constricted posteriad before neck constriction which is short (Figs 20–24), posterior supraorbital setae always present, whole body pubescent, covered with dense, erected short setae, anterior dorsal pore of 3^rd^ elytral stria absent (Fig. 7); ventrite VII in male with a pair of paramedian setae (Fig. 26) (subgenus *Dongodytodes* Tian)	**8**
2	Fore body extremely elongate especially on head, head (excluding mandibles) plus prothorax as long as elytra, propleura slightly expanded (Tian’e: cave Bahao Dong)	**Dongodytes (s. str.) giraffa Uéno**
–	Fore body less elongate, head (excluding mandibles) plus prothorax shorter than elytra, propleura strongly expanded	**3**
3	Posterior latero-marginal setae of pronotum absent	**4**
–	Posterior latero-marginal setae of pronotum present	**5**
4	Elytra pubescent, with three dorsal pores on 3^rd^ stria (Du’an: Lalie: cave Lapo Dong I)	**Dongodytes (s. str.) elongatus sp. n.**
–	Elytra smooth and glabrous, with two dorsal pores on 3^rd^ striae, the preapical one absent (Du’an: Longwan: cave Longhuan Dong)	**Dongodytes (s. str.) lani sp. n.**
5	Hind latero-marginal setae of pronotum distant from hind angles, pronotum glabrous, ventrites IV–VI each with two pairs of paramedian setae, median lobe of aedeagus distinctly curved at apex	**6**
–	Hind latero-marginal setae close to hind angles, pronotum covered by sparse and long setae, ventrites IV–VI each with only a pairs of paramedian setae, median lobe of aedeagus not curved at apex	**7**
6	Lateral borders of pronotum invisible from above in apical fifth, pronotum shorter (PL/PW=2.0) (Bama: cave Jiabao Dong)	**Dongodytes (s. str.) fowleri Deuve**
–	Lateral borders of pronotum visible from above in apical fifth, pronotum longer (PL/PW=2.1) (Fengshan: cave Yuanyang Dong)	**Dongodytes (s. str.) grandis Uéno**
7	Head and pronotum covered with sparser, long and erected setae, head broader, suddenly constricted before neck constriction (Fig. 16), hind angles of pronotum sharp, lateral sides of elytra near base nearly straight (Fig. 27) (Du’an: Chengjiang: cave Baxian Dong)	**Dongodytes (s. str.) baxian Tian**
	Head and pronotum covered with denser, long and erected setae, head elongate, gently and gradually narrowed before neck constriction (Fig. 19), hind angles of pronotum blunt, lateral sides of elytra near base slightly sinuate (Fig. 30) (Du’an: Longfu: cave Shuiyuan Dong)	**Dongodytes (s. str.) troglodytes sp. n.**
8	Body stout, pronotum with only a pair of latero-marginal setae close to hind angles	**9**
–	dle and a little before hind angles respectively	**10**
9	Head strongly expanded (Fig. 21), clypeus sexsetose, elytra elongate (Fig. 11) (Du’an: Gaoling: caves Jinzhu Dong I and Jinzhu Dong II)	**Dongodytes (Dongodytodes) jinzhuensis sp. n.**
–	Head elongate (Fig. 22), clypeus 10-setose, elytra more ovate (Fig. 12) (Gaoling: cave Nongguanshang Dong II)	**Dongodytes (Dongodytodes) inexpectatus sp. n.**
10	Head broader, sides nearly paralleled in median part (Fig. 20), lateral sides of pronotum almost straight or weakly sinuate before hind angles (Fig. 31) (Du’an: Xia’ao: cave Lubian Dong)	**Dongodytes (Dongodytodes) deharvengi Tian**
–	Head slender, sides not paralleled in median part, lateral sides of pronotum distinctly sinuate before hind angles	**11**
11	Small, propleura more expanded, elytra shorter, base of elytra broader (Figs 13–14), elytra with only preapical dorsal pore, median lobe of aedeagus stout and much shorter (Figs 43–44) (Du’an: Longwan: cave Nongqu Dong I; Chengjiang: caves Diaomao Dong and Nongzhong Dong I)	**Dongodytes (Dongodytodes) brevipenis sp. n.**
–	Large, propleura less expanded, elytra more elongate, base of elytra narrowed (Fig. 13), elytra with middle and apical dorsal pores, median lobe of aedeagus much slender (Figs 45–46) (Dahua: Qibainong: cave Qiaoxu Dong)	**Dongodytes (Dongodytodes) yaophilus sp. n.**

**Figures 6–7.**
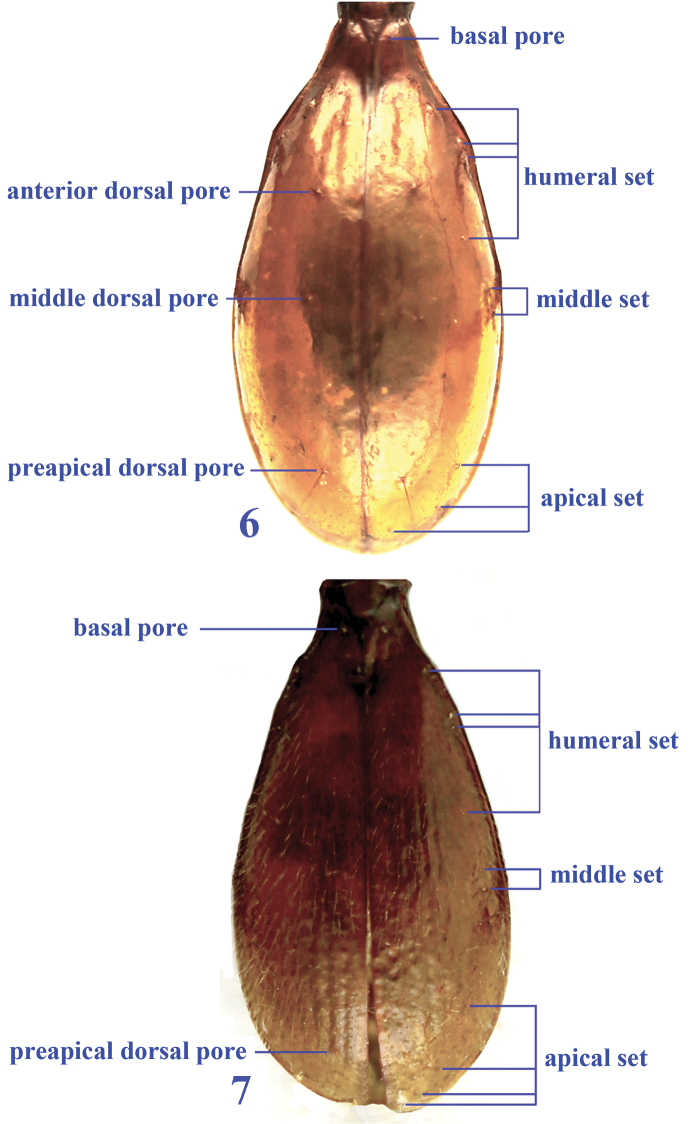
Elytral chaetotaxal patterns in *Dongodytes*. **6** Dongodytes (s. str.) lani sp. n. **7** Dongodytes (Dongodytodes) jinzhuensis sp. n.

**Figures 8–11.**
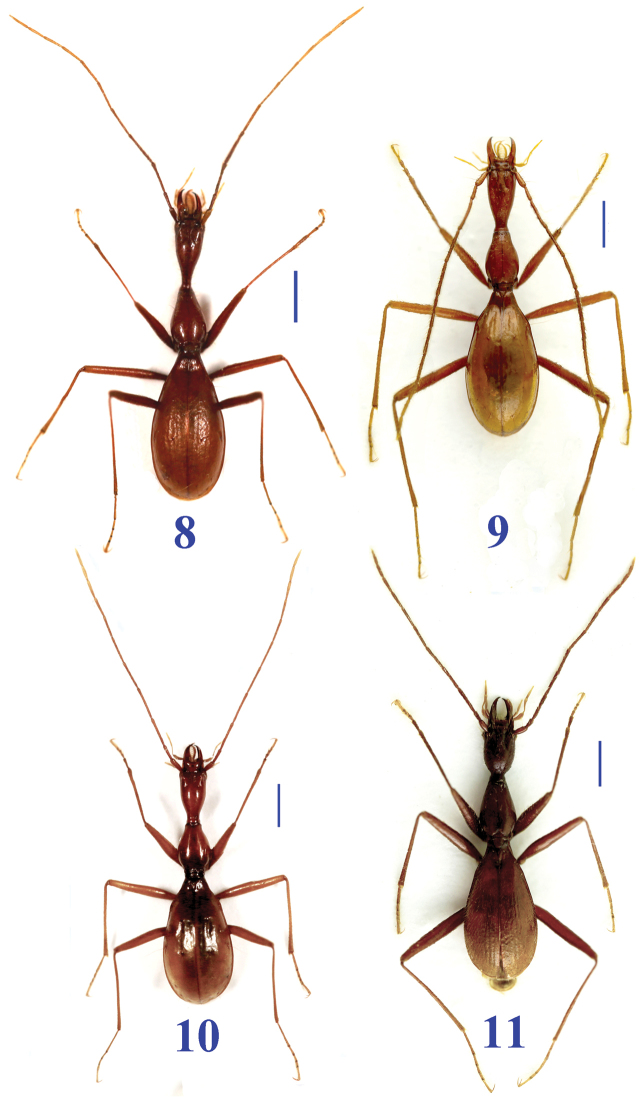
Habitus of *Dongodytes* species. **8** Dongodytes (s. str.) elongatus sp. n. female, paratype **9** Dongodytes (s. str.) troglodytes sp. n. male, holotype **10** Dongodytes (s. str.) lani sp. n. female, paratype **11** Dongodytes (Dongodytodes) jinzhuensis sp. n. female, paratype, from Jinzhu Dong I. Scale bar: 1.0 mm.

**Figures 12–15.**
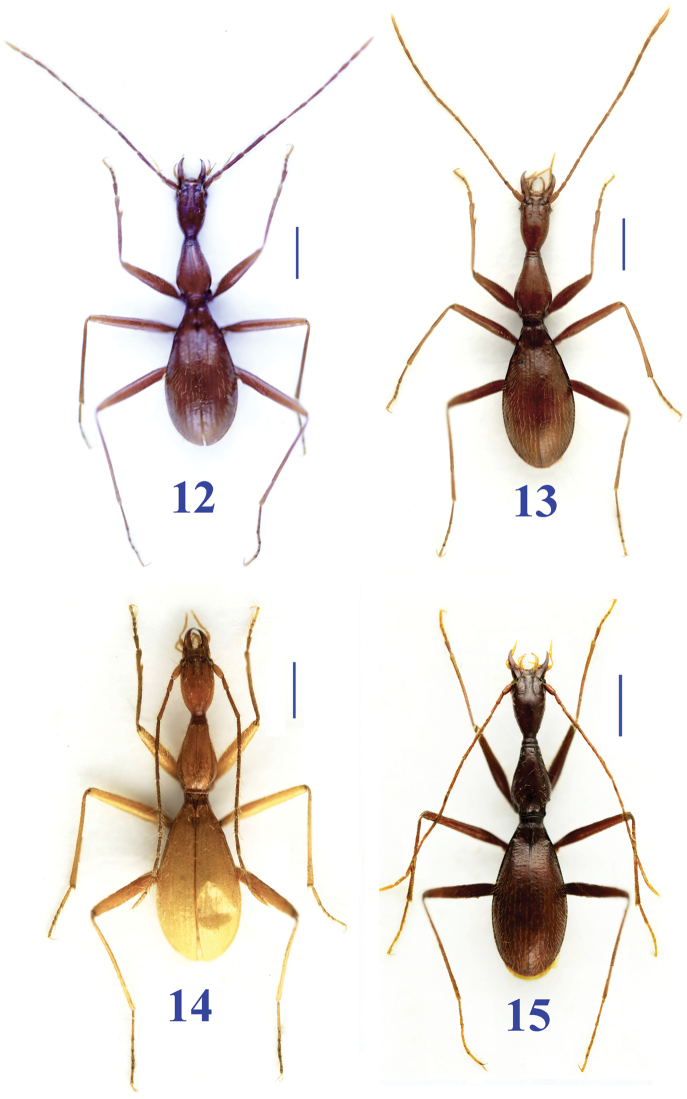
Habitus of *Dongodytes* species. **12** Dongodytes (Dongodytodes) inexpectatus sp. n. male, holotype **13** Dongodytes (Dongodytodes) brevipenis sp. n. male, paratype, from Longwan: Nongqu Dong **14** ibid, from Chengjinag: Diaomao Dong **15** Dongodytes (Dongodytodes) yaophilus sp. n. female, paratype. Scale bar: 1.0 mm.

**Figures 16–24.**
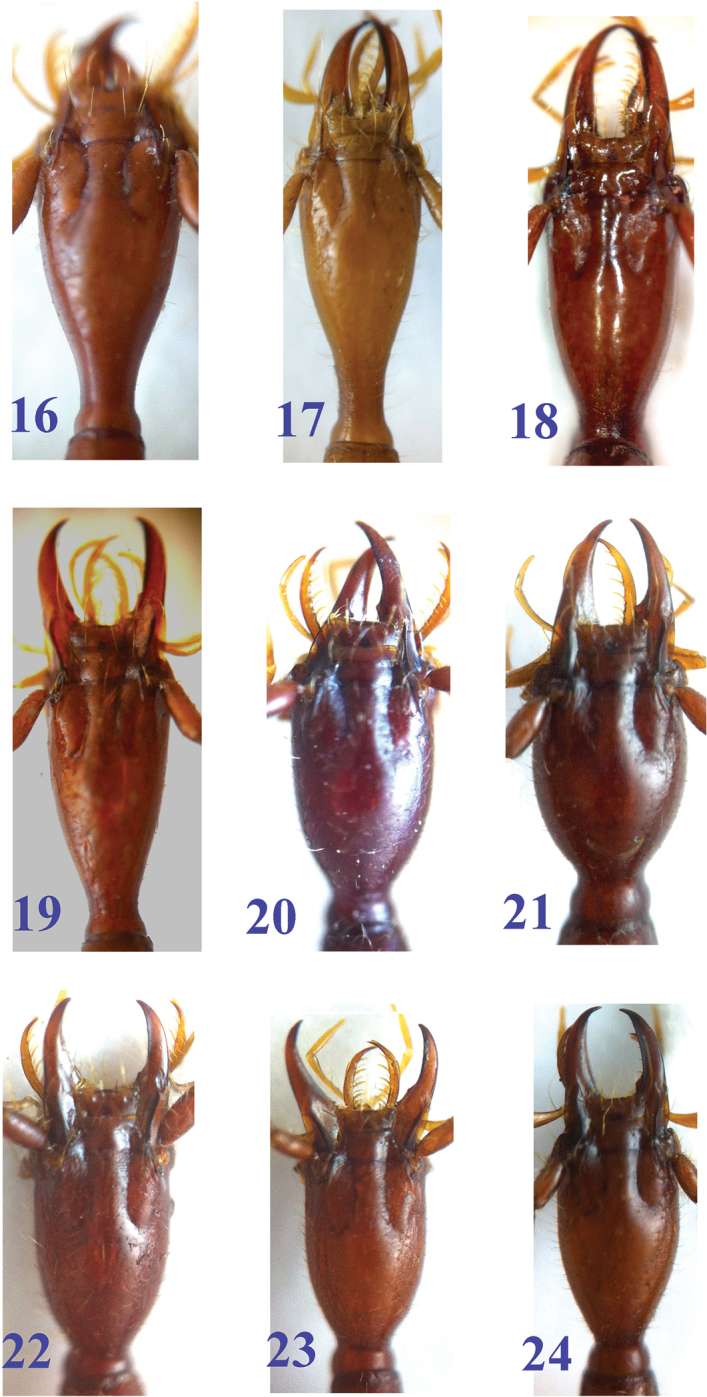
Head of *Dongodytes* species. **16** Dongodytes (s. str.) baxian M, male, holotype **17** Dongodytes (s. str.) elongatus sp. n. male, holotype **18** Dongodytes (s. str.) lani sp. n. male, holotype **19** Dongodytes (s. str.) troglodytes sp. n. male, holotype **20** Dongodytes (Dongodytodes) deharvengi, male, holotype **21** Dongodytes (Dongodytodes) jinzhuensis sp. n. female, paratype **22** Dongodytes (Dongodytodes) inexpectatus sp. n. male, holotype **23** Dongodytes (Dongodytodes) brevipenis sp. n. male, holotype **24** Dongodytes (Dongodytodes) yaophilus sp. n. male, holotype.

**Figures 25–26.**
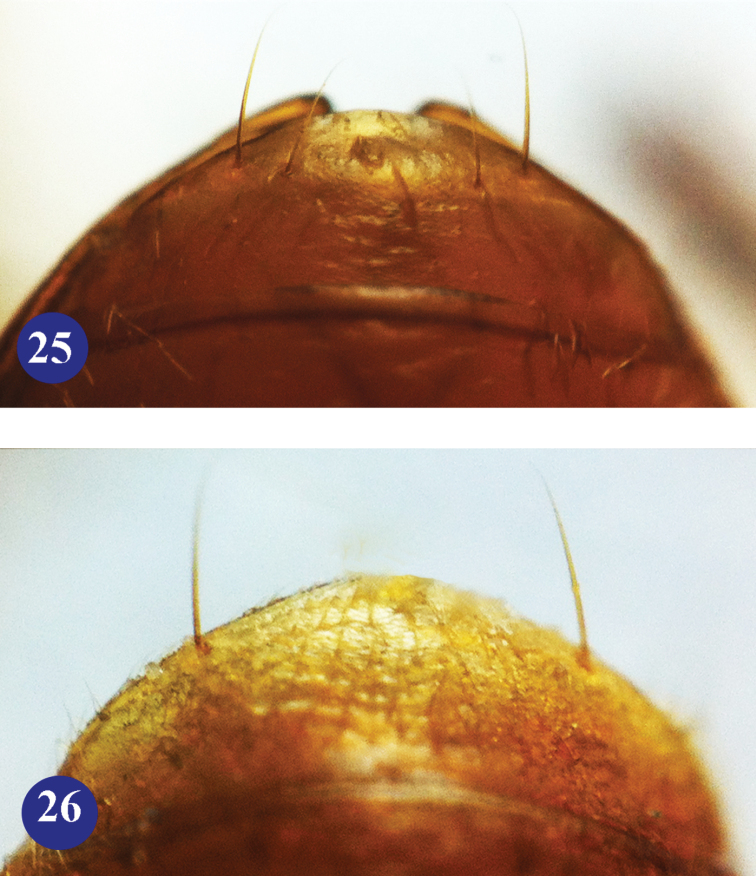
Abdominal ventrite VII of *Dongodytes* in male. **25** Dongodytes (s. str.) lani sp. n., paratype **26** Dongodytes (Dongodytodes) jinzhuensis sp. n., paratype.

**Figures 27–32.**
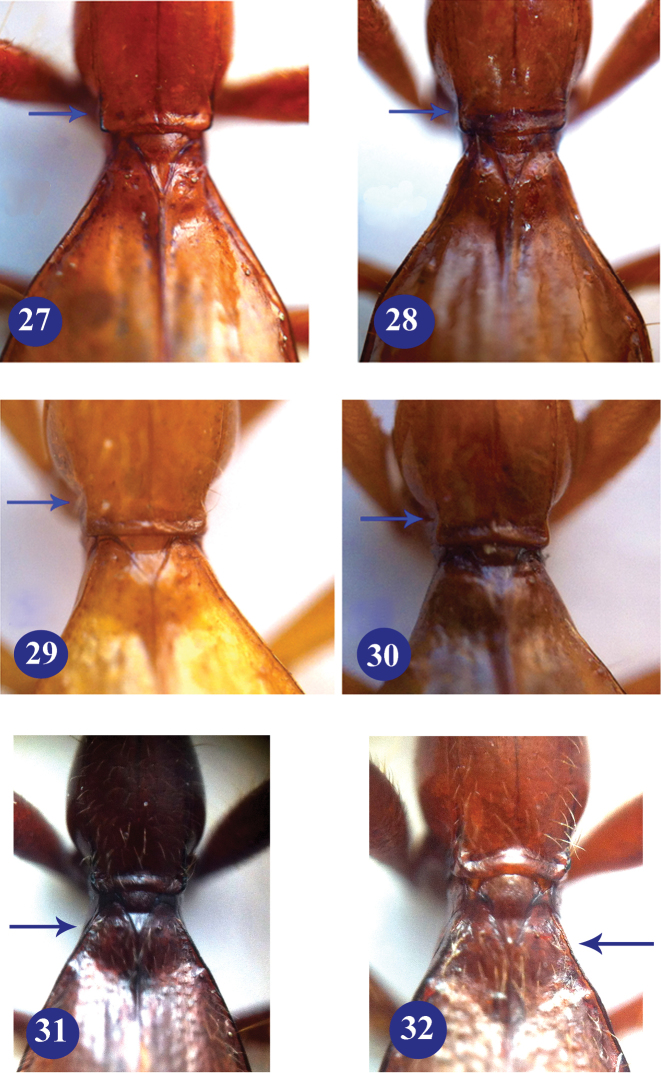
Basal parts of prothorax and elytra of subgenus *Dongodytes* (s. str.). **27** Dongodytes (s. str.) baxian, male, holotype **28** Dongodytes (s. str.) elongatus sp. n., male, holotype **29** Dongodytes (s. str.) lani sp. n., female, paratype **30** Dongodytes (s. str.) troglodytes sp. n., male, holotype **31** Dongodytes (Dongodytodes) deharvengi Tian, male, paratype **32** Dongodytes (Dongodytodes) jinzhuensis sp. n., female, paratype.

**Figures 33–38.**
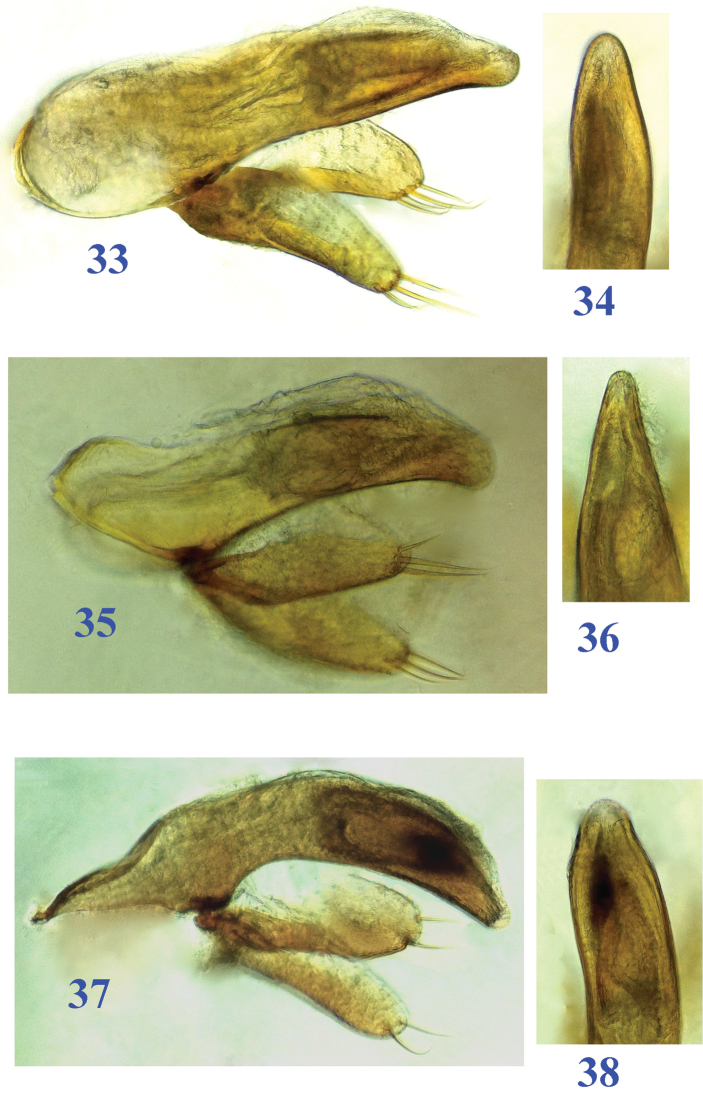
Male genitalia of *Dongodytes* (s. str.) species (median lobe and parameres in lateral view, apical part of median lobe in dorsal view). **33–34** Dongodytes (s. str.) elongatus sp. n. **35–36** Dongodytes (s. str.) lani sp. n. **37–38** Dongodytes (s. str.) troglodytes sp. n.

**Figures 39–46.**
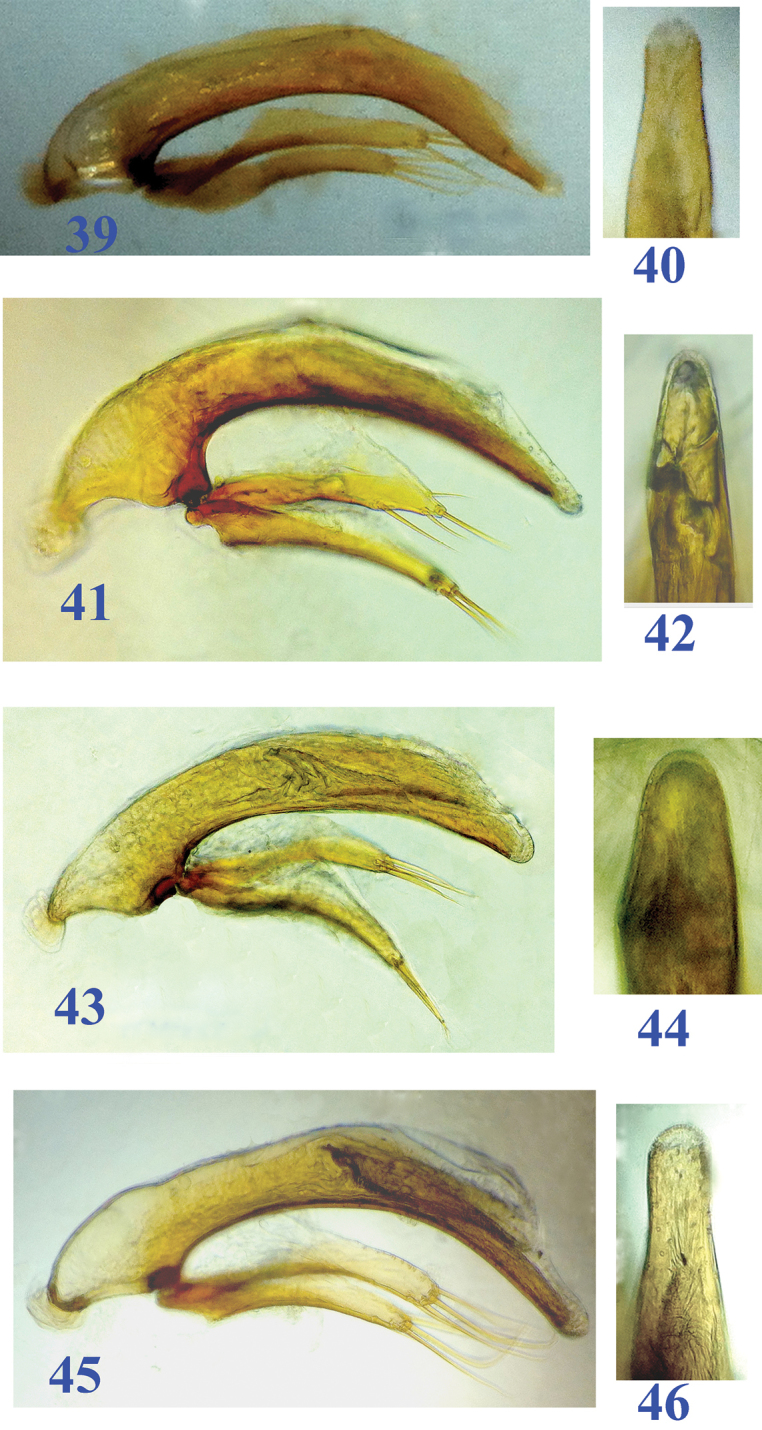
Male genitalia of Dongodytes (Dongodytodes) species (median lobe and parameres in lateral view, apical part of median lobe in dorsal view). **39–40** Dongodytes (Dongodytodes) jinzhuensis sp. n. **41–42** Dongodytes (Dongodytodes) inexpectatus sp. n. **43–44** Dongodytes (Dongodytodes) brevipenis sp. n. **45–46** Dongodytes (Dongodytodes) yaophilus sp. n.

### Subgenus Dongodytes (s. str.)

Taxon classificationAnimaliaColeopteraCarabidae

Deuve, 1993

Dongodytes Deuve, 1993: 292

#### Type species.

*Dongodytes fowleri* Deuve, 1993: 292.

#### Type locality:

Bama County: cave Jiabao Dong.

#### Diagnostic characters.

Body shiny and polished, antennae very long, extending over elytral apex, head elongate, genae gradually narrowed posteriad, not expanded medially, neck constriction long, posterior supraorbital setae present or not, head and pronotum glabrous or covered with sparse long and erected setae, elytra glabrous in most species (except for *Dongodytes elongatus* sp. n. which is wholly pubescent), anterior dorsal pore on 3^rd^ stria present (Fig. 6); legs very elongate, more slender than in *Dongodytodes*; ventrite VII in male with two pairs of paramedian setae in both sexes (Fig. 25).

#### Distribution.

Northwestern Guangxi (Du’an, Bama, Fengshan and Tian’e Counties) (Fig. 73). *Dongodytes* (s. str.) is composed of six species. Three of them are recorded from Du’an County, and a single species is known in each of Bama, Fengshan and Tian’e Counties, respectively. Each species of *Dongodytes* (s. str.) is only known from a single limestone cave.

### Dongodytes (s. str.) baxian

Taxon classificationAnimaliaColeopteraCarabidae

Tian, 2011

Figs 1f , 16 , 27 , 73j

Dongodytes (s. str.) baxian Tian, 2011: 62

#### Diagnosis.

Middle sized, head and pronotum sparsely covered with erected setae, elytra glabrous; head (Fig. 16) extremely elongate though comparatively stout, and gradually narrowed towards neck constriction; only anterior pair of supraorbital setae present; clypeus quadrisetose; head (excluding mandibles) plus prothorax distinctly shorter than elytra; pronotum with two pairs of latero-marginal setae, posterior ones at a little before hind angles; lateral borders near front angles clearly visible from above, hind angle rectangular and sharp (Fig. 27); 3^rd^ elytral stria with three dorsal pores, at about 2/7, 1/2 and 1/7 from base, respectively; marginal umbilicate pores: distance from 2^nd^ to 3^rd^ longer than half from 1^st^ to 2^nd^, distance from 4^th^ to 3^rd^ as long as that from 3^rd^ to 5^th^; male genitalia stout, nearly straight, base orifice very wide, with a small sagittal aileron, dorsally apical lobe broadly rounded; each paramere with four long setae at apex.

#### Material examined.

Only the male holotype (Tian 2011).

#### Distribution.

Guangxi (Du’an). Known only from the limestone cave called Baxian Dong, Chengjiang (Figs 1f and 73j).

Baxian Dong remains its natural state in some degree though it is located in Baxian Park close to Chengjiang. However, the species Dongodytes (s. str.) baxian is very rare. We visited the cave twice in June and December, 2013 respectively, but failed to collect any additional specimens. Major parts of the big cave are too dry, except for a small area at about 40 m from the upper entrance where water droppings create a suitable habitat for trechines.

### Dongodytes (s. str.) elongatus sp. n.

Taxon classificationAnimaliaColeopteraCarabidae

http://zoobank.org/9C648AB1-6334-44AA-B22F-966B67FDA7FD

Figs 1k , 8 , 17 , 28 , 33 –34 , 47 –49 , 73l

#### Description.

Length: 8.1–8.5 mm (mean 8.3 mm); width: 1.9–2.4 mm (mean 2.1 mm). Habitus as in Fig. 8.

Colour: Light yellowish brown to brown, dull, palps pale.

Macrosculpture: Head including underside surface and pronotum smooth and rather polish, sparsely covered with rather long and erected setae (except the tumid propleura), elytra and prosternum wholly covered with long setae; legs and abdominal ventrites pubescent.

Microsculpture: Engraved meshes clearly and strongly transverse on head and pronotum, faintly isodiametric on elytra.

Head (Fig. 17) much longer than wide, HL/HW=2.7–3.5 (mean 3.0); head excluding mandibles distinctly longer than pronotum, 1.3–2.3 times (mean 1.8), with a long and gradually narrowed neck constriction; widest at a little behind antennal articulations, two pairs of supraorbital setae present in holotype and three paratypes, setae on posterior pores shorter, asymmetrically sited, left one a little more behind than the right; right posterior pore absent in a female paratype; a pair of suborbital pores present, long, not far from the ring-shaped base, more or less asymmetrically sited; clypeus transverse, quadrisetose, sparsely covered with six additional short setae; labrum transverse, sexsetose; front shallowly emarginated; palps thin and very elongate, penultimate palpomeres longer than the apical ones; 2^nd^ labial palp bisetose on inner margin; mentum and submentum partly fused, labial suture shortly traceable at sides; mentum bisetose basally, mental tooth simple, mental pits fine but distinct; submentum octosetose (but 12-setose in one female paratype, and 13-setose in one male paratype); antennae filiform, extending over elytral apex, all antennomeres pubescent, 1^st^ as long as 2^nd^, 3^rd^ 1.84 times longer than 2^nd^, each of 3^rd^–5^th^ subequal in length, then gradually shortened towards apex, 10^th^ as long as 11^th^, slightly longer than 1^st^.

Prothorax comparatively short (though much longer than wide) and narrow, slightly wider than head, PW/HW=0.7–1.1 (mean 0.9); front much narrower than base, PAW/PBW=0.4–0.7 (mean 0.6); propleura strongly tumid, much wider than pronotum, PW/PTW=0.8–0.9 (mean 0.8); pronotum rather short, lateral borders invisible from above at about 1/6 of apical parts (but visible in a male paratype), hind angles (Fig. 28) nearly rectangular, but obtuse, posterior lateral setae absent.

Elytra very elongate (but abnormally ovate in a male paratype), EL/EW=1.8–2.1 (mean 2.0), much wider than prothorax, EW/PTW=2.0–2.1 (mean 2.0); moderately convex; base comparatively thick (Fig. 28), humeral parts nearly straight, apex round; widest at about 2/3 from base; elytra slightly longer than head (excluding mandibles) plus prothorax; striae shallow, 2^nd^ and 3^rd^ traceable, others vague; 3^rd^ elytral stria with three dorsal pores at about 1/3, 4/7 and 6/7 from base, respectively; chaetotaxal pattern of the marginal umbilicate pores similar in *Dongodytes baxian*, but distance from 1^st^ to 2^nd^ pores over twice as long as that from 2^nd^ to 3^rd^ (less than twice in *Dongodytes baxian*); distance from 3^rd^ to 4^th^ pores much longer than that from 4^th^ to 6^th^ (almost as long as in *Dongodytes baxian*).

Male genitalia (Figs 33–34): Median lobe of aedeagus stout, slightly sinuate before apex, apical part very short and broad, basal part wide and larger, basal orifice large, with margin distinctly protruding ventrally; ventral margin arcuate ventrad; sagittal aileron very small; inner sac armed with a broad and long copulatory piece which covered with scale structures on surface, as long as 1/3 of the median lobe; in dorsal, apical lobe very broad, and rounded at apex; parameres wide and rather long, right and left ones with four and three long apical setae respectively.

#### Remarks.

This species is a peculiar representative within *Dongodytes* (s. str.) because of its wholly pubescent elytra and comparatively shorter antennae in which only 10–11^th^ segments extending over elytra. It is rather similar to *Dongodytes baxian* in appearance, but easily distinguished from the latter by its larger body size, slenderer and more elongate head, more expanded of propleura, without posterior latero-marginal setae, and stouter aedeagus.

#### Etymology.

This new species is named referring to its very slender and elongate body.

#### Material examined.

Holotype: male, Guangxi: Du’an: Lalie: Fuyan: Jianong: cave Lapo Dong I, 24°11.987N, 108°20.378E, 140 m, 2013-VI-23, leg. Mingyi Tian, Weixin Liu, Haomin Yin & Sunbin Huang; Paratypes: 2 males and 2 females, ibid. All are deposited in SCAU.

#### Distribution.

Guangxi (Du’an). Known only from the type locality, cave Lapo Dong I in Lalie (Figs 1k and 73l).

**Figures 47–49.**
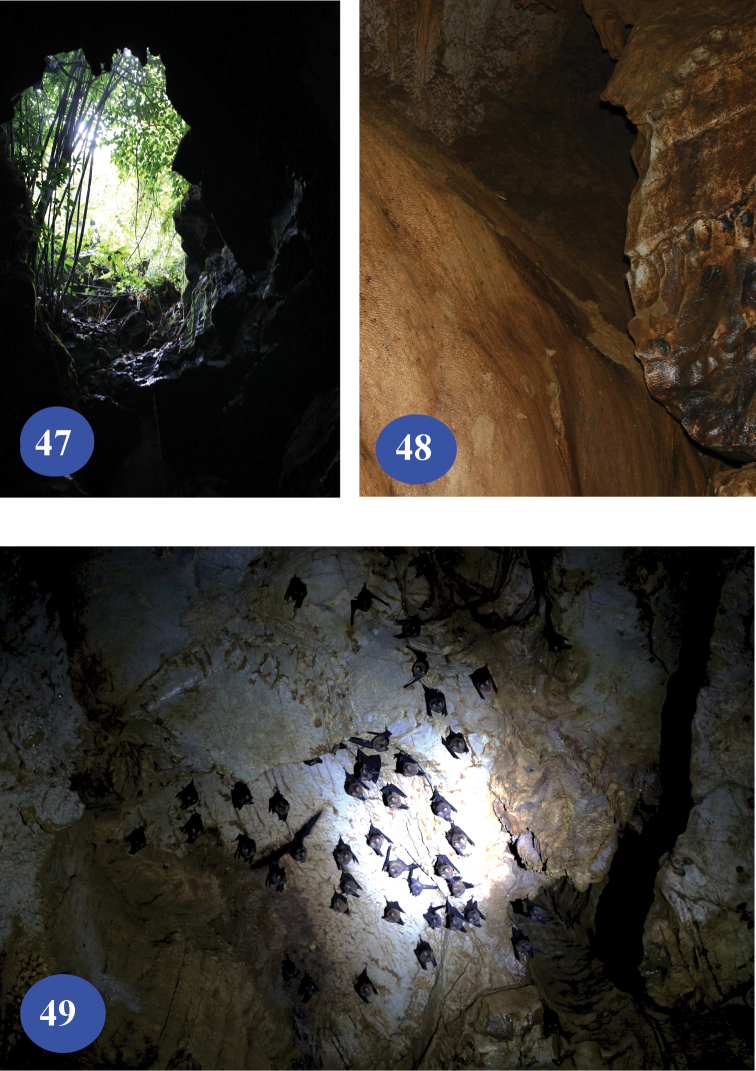
Lapo Dong I, type locality of Dongodytes (s. str.) elongatus sp. n. **47** entrance **48** wall of the cave, to show where the beetles were collected **49** bats on roof of the cave.

The species was found close to the entrance of Lapo Dong (Figs 47–49). Deeper parts of the cave were not accessible during our visit. It was said that the cave is about 400 m long. There is no pool in the cave, but it is wet and muddy, and covered with guano. Other cave-dwelling animals living in this cave are crickets, mosquitoes, pselaphids, spiders, millipedes, snails and bats.

### Dongodytes (s. str.) troglodytes sp. n.

Taxon classificationAnimaliaColeopteraCarabidae

http://zoobank.org/63074B01-10B2-4531-96D3-90DECF2DAB95

Figs 1c , 9 , 19 , 30 , 37 –38 , 50 –52 , 73f

#### Description.

Length: 7.0–7.5 mm (mean 7.3 mm); width: 1.9–2.4 mm (mean 2.1 mm). Habitus as in Fig. 9.

Colour: Light yellowish brown, palps pale.

Macrosculpture: Surface smooth, polish and strongly shiny; head including underside surface and prothorax sparsely covered with rather long and erected setae except the tumid propleura, elytra glabrous; prosternum with a row of 6–8 setae on each side; legs and abdominal ventrites pubescent.

Microsculpture: Engraved meshes faint, densely and strongly transverse on head and pronotum, without clear meshes on elytra.

Head (Fig. 19) nearly reverse triangular shaped, much longer than wide, HL/HW=3.1–3.7 (mean 3.4); slightly less elongate than in *Dongodytes elongatus* sp. n., but more slender than in *Dongodytes baxian*; head excluding mandibles slightly longer than pronotum, with mandibles much longer than pronotum (1.9–2.0 times, mean 1.9); gradually narrowed posteriad, forming a collar-like neck, neck constriction distinct, about half as wide as head; two pairs of supraorbital setae present, at about 1/3 and 2/3 from apex respectively; setae of anterior pores long and distinct, of posterior pores short and indistinct, only slightly longer than other erected setae nearby; frontal furrows short, deeply impressed and narrow; clypeus transverse, sexsetose; labrum similar in *Dongodytes baxian*, but less emarginated apically; palps thin and very elongate, 3^rd^ maxillary palpomere as long as 4^th^; 2^nd^ labial palpomere distinctly longer than 3^rd^, and bisetose on inner margin; mentum and submentum not fused, labial suture distinct; mentum bisetose basally, mental tooth simple, blunt apically, a pair of basal pits small but distinct; submentum octosetose but 12-setose in one female paratype, and 14-setose in one male paratype; antennae filiform, wholly pubescent, extending over elytral apex, as long as in *Dongodytes baxian*; 1^st^ antennomere as long as 2^nd^, 10^th^, and 11^th^, respectively, 3^rd^ longest, about twice as long as 2^nd^, then gradually shortened towards apex.

Prothorax short though much longer than wide and narrow, slightly wider than head, propleura strongly tumid, PW/PTW=0.8, front narrower than base, PAW/PBW=0.7–0.8 (mean 0.8); pronotum narrower than head, PW/HW=0.8–1.0 (mean 0.9); lateral borders of pronotum invisible from above at 1/6 of apical parts (but visible in a male paratype); hind angles nearly rectangular, but obtuse (Fig. 30), posterior latero-marginal setae present, with location as in Dongodytes (s. str.) baxian.

Elytra very elongate, much longer than wide, EL/EW=1.8–2.0 (mean 2.0); slightly longer than head (excluding mandibles) plus prothorax, twice as wide as prothorax, EW/PTW=2.0–2.2 (mean 2.1); base thin (Fig. 30); humeral parts slightly and widely sinuate, apex round; widest at about 3/5 from base; disc strongly convex; striae shallow and vague though 2^nd^ and 3^rd^ traceable; 3^rd^ stria with three dorsal pores at about 1/3, 1/2 and 5/6 from base, respectively; chaetotaxal pattern of marginal umbilicate pores similar in *Dongodytes baxian*.

Male genitalia (Figs 37–38): Median lobe of aedeagus short and rather slender, ventral margin strongly arcuate, not sinuate before apex, bisinuate dorsally; apical part short and blunt, basal part very wide and long, basal orifice very large; sagittal aileron very small; inner sac armed with a broad and long copulatory piece which covered with scale structures on surface, about 1/3 as long as the median lobe; in dorsal, apical lobe very broad, and more rounded at apex than other species; parameres wide and rather long, each of right and left parameres with two long setae at apex.

#### Remarks.

*Dongodytes troglodytes* sp. n. is similar to *Dongodytes baxian*. It differs from the latter by the following characters: head more elongate, narrower, gently and gradually constricted towards neck constriction (stouter and quickly constricted before neck constriction in *Dongodytes baxian*); hind angles of pronotum blunt (sharp in *Dongodytes baxian*); head and pronotum covered with denser setae (sparser in *Dongodytes baxian*); elytra narrower, but more convex (broader but rather flat in *Dongodytes baxian*); and marginal borders at humeral parts slightly sinuate (nearly straight in *Dongodytes baxian*).

#### Etymology.

Referring to its cave-adapted morphological characters.

#### Materials examined.

Holotype: male, Guangxi: Du’an: Longfu: Shangme: cave Shuiyuan Dong, 24°11.335N, 107°49.865E, 509 m, 2013-VI-28, leg. Mingyi Tian, Wei Lin, Weixin Liu, Haomin Yin & Sunbin Huang, in SCAU; Paratypes: 9 males, 22 females, ibid; 11 females, 2013-V-2, leg. Mingyi Tian, Weixin Liu, Feifei Sun & Haomin Yin, in SCAU, MNHN, IOZ and ZUBM, respectively.

#### Distribution.

Guangxi (Du’an) (Fig. 73f). Known only from the type locality, a cave called Shuiyuan Dong in Longfu (Figs 1c and 73f).

**Figures 50–52.**
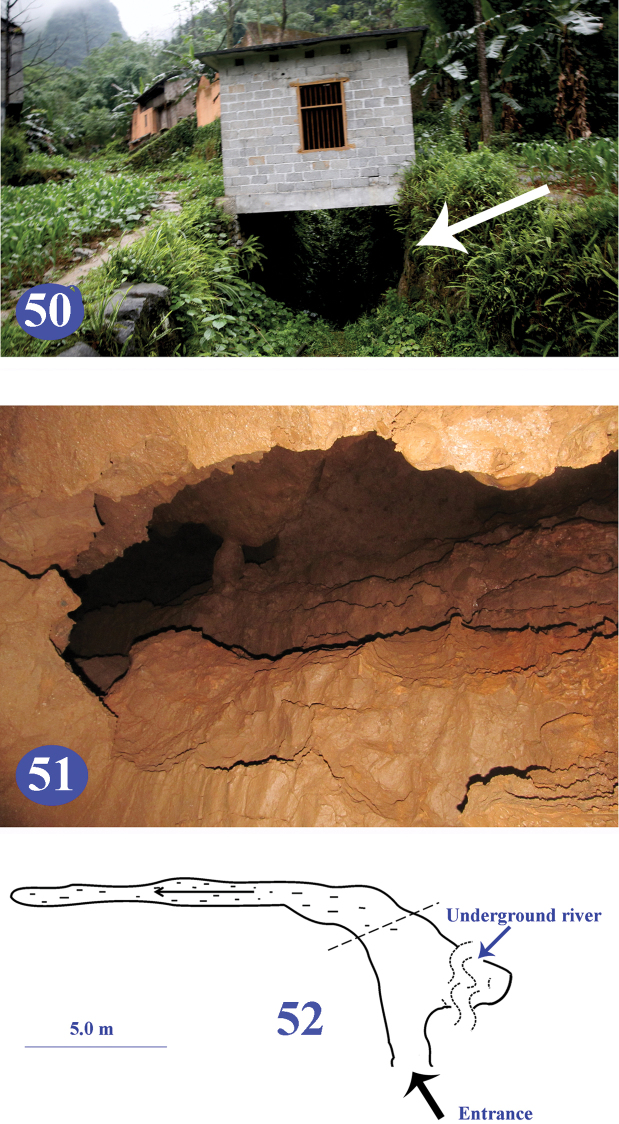
Shuiyuan Dong in Longfu, type locality of Dongodytes (s. str.) troglodytes sp. n. **50** entrance (indicated by arrowhead) **51** passage of upper layer where the beetles were collected **52** a sketch of the cave.

Shuiyuan Dong (Figs 50–52) is composed of two layers. The lower layer is an underground river which provides water source for the local people and it is impossible to entre. The upper part is a short passage, one to one and half metres high, two to four metres wide, and about 15 m long. All of the specimens of *Dongodytes troglodytes* sp. n. were collected in the upper part of the cave. Apart from *Dongodytes*, another anophthalmic trechine, belonging to the genus *Libotrechus* Uéno, 1998, was also collected in the cave (Lin and Tian 2014). Other cave animals found in Shuiyuan Dong are crickets, millipedes, isopods, moths, spiders, mosquitoes, snails, bats and cave fishes.

### Dongodytes (s. str.) lani sp. n.

Taxon classificationAnimaliaColeopteraCarabidae

http://zoobank.org/9F7B7024-FF9F-45E3-B775-ECBE05133275

Figs 1j , 6 , 10 , 18 , 25 , 29 , 35 –36 , 53 –55 and 73k

#### Description.

Length: 6.8–7.3 mm (mean 7.1 mm); width: 1.9–2.1 mm (mean 2.0 mm). Habitus as in Fig. 10.

Colour: Light yellowish brown, palps pale.

Macrosculpture: Surface smooth, polish and strongly shiny; head including underside surface and pronotum sparsely covered with rather long and erected setae (except the tumid propleura), elytra glabrous; prosternum with a row of 4–5 setae on each side; legs and abdominal ventrites pubescent.

Microsculpture: Engraved meshes densely and strongly transverse on head and pronotum, less transverse and faint on elytra.

Shape of the head (Fig. 18) intermediate between *Dongodytes* (s. str.) and *Dongodytodes*, genae more elongate than in *Dongodytodes* but more expanded posteriorly than in other *Dongodytes* (s. str.), widest at middle part; much longer than wide, HL/HW=3.0–3.5 (mean 3.2), excluding mandibles distinctly longer than pronotum (1.6–1.8 times, mean 1. 7); neck constriction shorter but broader, about half as wide as head; two pairs of supraorbital setae present, at about 1/3 and 2/3 from apex respectively; setae of anterior pores long and distinct, of posterior pores short and indistinct, slightly longer than other erected setae nearby, and asymmetrically sited; frontal furrows short, deeply impressed and narrow, not parallel medially; clypeus transverse, sexsetose; labrum transverse, distinctly emarginated apically; a pair of suborbital setae present; palps thin and very elongate, 3^rd^ maxillary palpomere slightly longer than 4^th^; 2^nd^ labial palpomere distinctly longer than 3^rd^, and bisetose on inner margin; ligula bisetose, but multi-setose at apex with adnated paraglosae; mentum and submentum well separated by clear labial suture; mentum bisetose medially, mental tooth simple, broad apically, a pair of basal pits small but distinct; submentum octosetose; antennae wholly pubescent, as long as in *Dongodytes baxian*, extending over elytral apex; 1^st^ antennomere distinctly longer than 2^nd^ which is as long as 10^th^ and shorter than 11^th^, 3^rd^ as long as 5^th^ and slightly shorter than 4^th^ which is the longest, 3^rd^ nearly twice as long as 2^nd^, from 5^th^ gradually shortened towards 10^th^.

Prothorax short and stout though much longer than wide, distinctly wider than head, widest at about 2/5 from base; propleura strongly tumid; PAW/PBW=0. 7–0.9 (mean 0.8), PW/PTW=0.7–0.8 (mean 0.7); pronotum narrow, slightly narrower than head, PW/HW=0.8–1.0 (mean 0.9), lateral borders invisible from above at about 1/6 of apical parts; widest at about middle, as wide as head; only a pair of latero-marginal setae present (posterior ones absent), at about middle, lateral sides strongly sinuate before hind angles which are bluntly acute (Fig. 29).

Elytra (Fig. 6) very elongate ovate, almost as long as head (excluding mandibles) plus prothorax; EW/PTW=2.0–2.2 (mean 2.1), EL/EW=1.9; base thin, sides at humeral parts slightly and widely sinuate, forming very faint shoulders (Fig. 29), where borders indistinct; apex rounded; comparatively long, distinctly longer than head (including mandibles) plus prothorax, widest at about 2/3 from base; striae shallow and vague, though 1^st^ and 2^nd^ are traceable; two dorsal pores present on areas of 3^rd^ stria, at about 1/3 and 4/7 from base respectively; preapical dorsal pore absent; chaetotaxal pattern of marginal umbilicate pores similar in *Dongodytes baxian*, but 1^st^ pore of the humeral set closer to marginal gutter, and far from 2^nd^, distance of them three times as long as from 2^nd^ and 3^rd^.

Male genitalia (Figs 35–36): Median lobe of aedeagus very short, distinctly stouter than that of *Dongodytes baxian* and *Dongodytes elongatus* sp. n., ventral margin slightly and gently arcuate, slightly sinuate before apex which is broadly rounded, basal part very wide and long, nearly straight ventrally, basal orifice large; sagittal aileron very small and indistinct; inner sac armed with a large and long copulatory piece which covered with scale structures on surface, about 2/5 as long as the median lobe; in dorsal, apical lobe is slender, distinctly narrower than other congeners; parameres wide and broad, each of right and left parameres with three long apical setae respectively.

#### Remarks.

*Dongodytes lani* sp. n. is also a peculiar species in *Dongodytes* (s. str.) by having peculiar structures of head and aedeagus. Compared to *Dongodytes elongatus* sp. n. for which the posterior latero-marginal setae on pronotum are also lacking, *Dongodytes lani* sp. n. has stouter but shorter body, smooth and glabrous elytra, its genae more expanded posteriorly, and elytra without preapical dorsal pore (present in *Dongodytes elongatus* sp. n.).

#### Etymology.

Dedicated to Prof. Jiahu Lan (Du’an Fishery Technique Popularization Station, Guangxi), a well-known cave fish specialist in China, for thanking his various assistances and efficient cooperation during our biospeleological surveys in Du’an Karst.

#### Material examined.

Holotype: male, Guangxi: Du’an: Longwan: cave Longhuan Dong, 23°49.5213N, 108°14.4792E, 248 m, 2013-XII-24, leg. Mingyi Tian, Weixin Liu, Haomin Yin & Xiaozhu Luo, in SCAU; Paratypes: 1 male and 4 females, ibid, in SCAU.

#### Distribution.

Guangxi (Du’an). Known only from the type locality, a cave called Longhuan Dong in Longwan (Figs 1j and 73k).

**Figures 53–55.**
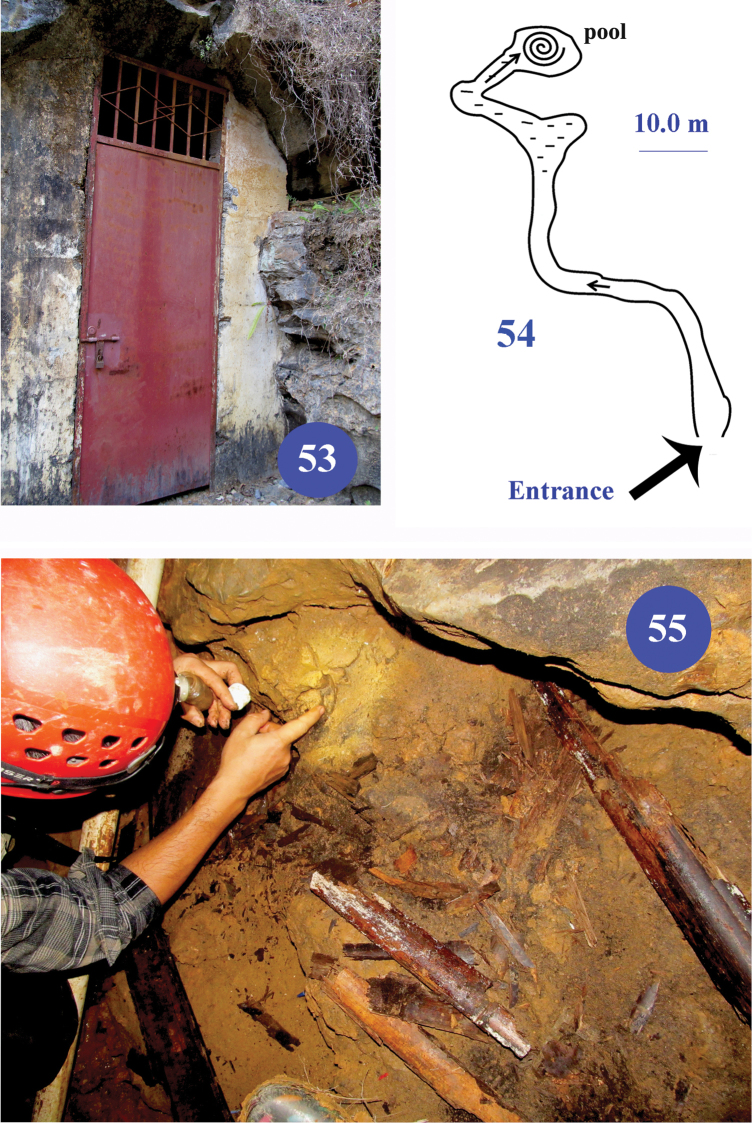
Longhuan Dong, type locality of Dongodytes (s. str.) lani sp. n. **53** entrance of the cave **54** a sketch of the cave **55** picture to show where the type series were collected in the cave.

Longhuan Dong (Figs 53–55) is about 100 m long, one to two metres wide and one to two metres high. There is a pool at the end of the passage, which is the water source for the local people. Part of the main passage is an artificial tunnel and very dry. The blind beetles were collected under pieces of decayed woods in a wet area just close to the pool. Other animals living in the cave are crickets, diplurans, isopods, millipedes, scutigers and snails.

### Subgenus Dongodytodes

Taxon classificationAnimaliaColeopteraCarabidae

Tian, 2011

Dongodytes (Dongodytodes) Tian, 2011: 57.

#### Type species.

Dongodytes (Dongodytodes) deharvengi Tian, 2011.

#### Type locality.

a limestone cave called Lubian Dong in Xia’ao, Du’an.

#### Diagnostic characters.

Body pubescent, covered with dense, erected short pubescence; antennae short, not extending over elytral apex; head stout and expanded posteriorly, suddenly constricted before neck constriction which is very short, posterior supraorbital setae always present; anterior dorsal pore on 3^rd^ elytral stria absent, preapical pore present (Fig. 7); legs thin and elongate, but slightly stouter than in *Dongodytes* (s. str.), ventrite VII in male with a pair of paramedian setae (Fig. 26).

#### Distribution.

Northwestern Guangxi (Du’an and Dahua Counties) (Fig. 73). *Dongodytodes* is composed of five species, all of them are occurring in Du’an Karst, and at the eastern (or northern) bank of the river Hongshui He. Each species is recorded from a single cave except *Dongodytes jinzhuensis* sp. n. (from two caves which are close to each other in Gaoling) and *Dongodytes brevipenis* sp. n. (from three caves which are very close to one another in Longwan and Chengjiang).

### Dongodytes (Dongodytodes) deharvengi

Taxon classificationAnimaliaColeopteraCarabidae

Tian, 2011

Figs 1b , 20 , 31 , 73e

#### Diagnosis.

Small sized, dark reddish brown, whole surface covered with dense and short bristly setae; head stout though rather elongate (Fig. 20), head excluding mandibles slightly longer than prothorax; genae distinctly expanded posteriorly, then suddenly constricted before the collar-shaped neck, posterior supraorbital setae present and close to neck constriction; mentum and submentum well separated by labial suture; antennae long, extending at about apical 1/6 of elytra; clypeus transverse, sexsetose; prothorax much longer than wide though propleura distinctly tumid, widest at about 1/3 from base; pronotum narrow, lateral borders almost parallel-sided but feebly expanded at about 1/3 basally, slightly sinuate before hind angles (Fig. 31); front angles right, hind angles obtuse; two pairs of latero-marginal setae present, at about 2/5 from base and a little before hind angles respectively; elytra as long as head with mandibles and pronotum combined, widest at about apical 2/5 of elytra, lateral sides near base straight (Fig. 31); two (middle and preapical) dorsal pores present on 3^rd^ elytral stria, at about middle and apical 1/6 respectively; median lobe of aedeagus slender, widely and evenly arcuate, sagittal aileron moderately sized, each of parameres with four long apical setae.

#### Material examined.

Apart from the type series (Tian 2011), two additional specimens were studied: 1 male and 1 female, cave Lubian Dong, same cave of the type locality, 310 m, Du’an: Xia’ao, 2013-V-02, leg. Mingyi Tian, Weixin Liu, Feifei Sun and Haomin Yin, in SCAU.

#### Distribution.

Guangxi (Du’an). Known only from the cave called Lubian Dong which was pointed out as an anonymous cave in the original description (Tian 2011), in Xia’ao, northern Du’an (Figs 1b and 73e). We visited this cave four times in 2013, but collected only two specimens more during the first visit in early May. The reason is probably that the beetle’s habitats had been partly changed after the huge collapse in the cave during the summer raining season.

### Dongodytes (Dongodytodes) brevipenis sp. n.

Taxon classificationAnimaliaColeopteraCarabidae

http://zoobank.org/1D24FECA-BB71-4A22-979E-593FE59F5BB2

Figs 1j, h, i , 13 –14 , 23 , 43 –44 , 56 –60 , 73i

#### Description.

Length: 6.8–7.1 mm (mean 6.9 mm); width: 1.7–2.0 mm (mean 1.8 mm). Habitus as in Figs 13–14, and 58–60.

Colour: Yellowish to light dark brown, palps pale.

Macrosculpture: Head and pronotum smooth, elytra vaguely punctate, body moderately shiny and wholly pubescent.

Microsculpture: Similar in *Dongodytes deharvengi*.

Head (Fig. 23) elongate, much longer than wide, HL/HW=2. 7–3.0 (mean 2.9); widest at about middle (from labrum), excluding mandibles as long as prothorax, including mandibles much longer than pronotum (1.4–1.6 times, mean 1.5); head (including mandibles) plus prothorax as long as elytra; head rather slender, genae less expanded laterally and posteriad, nearly parallel-sided medially; collar-shaped neck distinct, about 3/7 as wide as head; two pairs of supraorbital setae present, anterior ones far from each other, posterior ones close to each other; a pair of suborbital setae present, a pair of mental pits present; mental tooth bluntly bifid at apex; 2^nd^ labial palpomere bisetose on inner margin; clypeus transverse, sexsetose; ligula multi-setose; mentum and submentum fused partly, labial suture indistinct in median part; mentum sexsetose, apart from a pair of median setae beneath mental tooth, a pair of long basal setae present, and each accompanied by a short seta at front; submentum 10-setose; antennae extending at about apical 1/6 of elytra; 1^st^ antennomere strongly dilated, much more wider than others, distinctly longer than 2^nd^ which is the shortest, 3^rd^ longest, about twice as long as 2^nd^, and slightly longer than 4^th^, then gradually shortened towards 10^th^ which is slightly longer than 1^st^ but shorter than 11^th^.

Prothorax with propleura strongly tumid, widest at about 1/3 from base; base much wider than front, PAW/PBW=0.7–0.8 (mean 0.7); pronotum much narrower than prothorax, PW/PTW=0.7–0.8 (mean 0.8), and narrower than head, PW/HW=0.8–1.0 (mean 0.9), with two pairs of latero-marginal setae at a little before middle and a little before hind angles respectively, lateral sides distinctly sinuate in front of hind angles.

Elytra moderately elongate ovate, twice as long as wide, EL/EW=2.0–2.1 (mean 2.0); widest at about 2/3 from base; elytral base narrow, with sides almost straight; about twice as wide as prothorax, EW/PTW=2.0–2.1 (mean 2.0); striae well defined and punctate, only preapical dorsal pore present, at about 1/7 from apex; chaetotaxy of marginal umbilicate pores similar in *Dongodytes deharvengi*, but 4^th^ pore of humeral set is a little farer from 3^rd^.

Male genitalia (Figs 43–44): Median lobe of aedeagus very short, ventral margin gently arcuate, sagittal aileron distinct, basal orifice wide, apical part very short and bluntly rounded at apex; inner sac armed with a slender copulatory piece which covered with scale structures on surface, about 1/3 as long as the median lobe; in dorsal view, apical lobe very wide, much broader than other species of *Dongodytodes*; parameres moderately developed, right and left ones each with three and four long setae at apex respectively.

#### Remarks.

Differs from other congeners by its slender body, genae nearly parallel-sided at middle, sexsetose mentum, unique dorsal pore on 3^rd^ elytral stria, and very short aedeagus.

#### Etymology.

The name of this new species refers to its short aedeagus.

#### Material examined.

Holotype: male, Guangxi: Du’an: Longwan: cave Nongqu Dong I, 23°56.021N, 108°10.962E, 459 m, 2013-VI-27, leg. Mingyi Tian, Wei Lin, Weixin Liu, Haomin Yin & Sunbin Huang, in SCAU; Paratypes: 2 males, 5 females, ibid; 1 male, Guangxi: Du’an: Chengjiang: Ganwan: cave Nongzhong Dong I, 23°56.644N, 108°10.072E, 469 m, 2013-VI-27, same collectors as above; 13 males, 7 females, Guangxi: Du’an: Chengjing: Wanmao: cave Diaomao Dong, 24°01.723N, 108°07.236E, 140 m, 2013-VI-23, same collectors as above, in SCAU, IOZ, MNHN and ZUBM, respectively.

#### Distribution.

Guangxi (Du’an). Known only from the type localities, cave Nongqu Dong in Longwan, and caves Diaomao Dong and Nongzhong Dong I in Chengjiang (Figs 1g, h, i and 73i).

The entrance of Nongqu Dong (Fig. 57) is localized on the bottom of a hill and close to a corn field surrounded by trees. It is about 300 m long, with a lot of water droppings, and a small stream inside. It is a beautiful cave, with magnificent stone pillars, stalactites and stalagmites.

**Figures 56–60.**
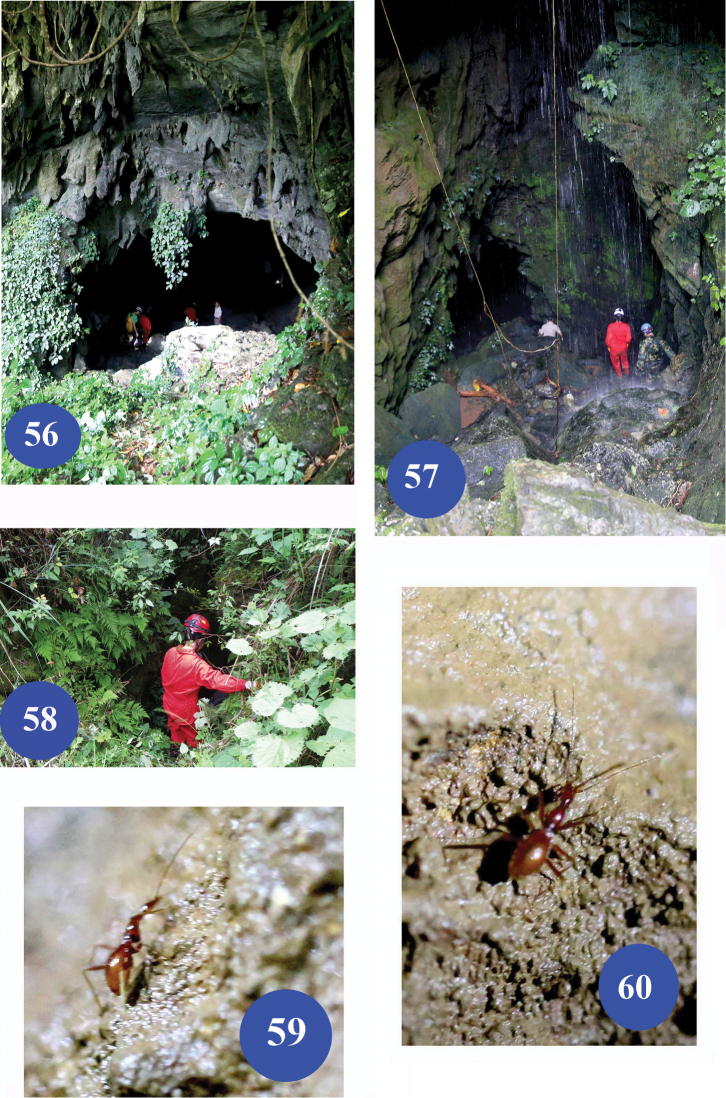
Type locality caves of Dongodytes (Dongodytodes) brevipenis sp. n. **56** entrance of Nongzhong Dong I **57** entrance of Nongqu Dong I **58** entrance of Diaomao Dong **59–60** beetles running on walls in Diaomao Dong.

The entrance of Nongzhong Dong I (Fig. 56) is in a sugarcane field. It is about 80 m long, composed of a main passage and several small galleries. At the end of the main passage there is an underground river which is about two kilometres long said by the local people.

Diaomao Dong I (Fig. 58) is localized at the foot of a hill near the village Nongzhong. It’s length is about 100 m. The entrance is surrounded by bamboos. The cave is wet, with several small pools and a stream.

The trechine beetles were collected in several places of dark areas in the caves. Other animals living in the above caves are crickets, mosquitoes, staphylinids, millipedes, centipedes, isopods, harvestmen, spiders, snails and cave fishes.

### Dongodytes (Dongodytodes) jinzhuensis sp. n.

Taxon classificationAnimaliaColeopteraCarabidae

http://zoobank.org/CBEB2BCE-5B76-45F7-871B-B7FA2AC7EA0A

Figs 1d , 7 , 11 , 21 , 26 , 32 , 39 –40 , 61 –65 , 73g

#### Description.

Length: 7.0–7.1 mm (mean 7.0 mm); width: 1.8–1.9 mm (mean 1.8 mm). Habitus as in Fig. 11.

Colour: Light dark brown, palps pale.

Macrosculpture: Head and pronotum smooth, elytra vaguely punctate, body moderately shiny and wholly pubescent.

Microsculpture: Densely and irregularly striate on head, pronotum and elytra.

Head (Fig. 21) very stout though elongate, distinctly longer than wide, HL/HW=2.6–2.8 (mean 2.7); widest at about middle from labrum, excluding mandibles as long as pronotum; head (including mandibles) plus prothorax as long as elytra; genae extremely dilated, making head much broader than any other species of the subgenus, about 2.7 times wider than collar-shaped neck, suddenly constricted before collar-shaped neck; nearly parallel-sided in median part; clypeus sexsetose; two pairs of supraorbital setae present, anterior setae distinctly longer than the posterior ones; a suborbital setae present, close to neck constriction; 2^nd^ labial palpomere much longer than 3^rd^, bisetose on inner margin; ligula multi-setose apically; labial suture faintly traceable, mentum with two pairs of setae, at base and median parts respectively, a pair of mental pits present; mental tooth bluntly bifid at apex; submentum 10-setose; antennae short, extending at about apical 1/6 of elytra; 1^st^ antennomere dilated, slightly longer than 2^nd^ which is the shortest, 3^rd^ the longest, twice as long as 2^nd^, gradually shortened from 4^th^ towards 10^th^ which is slightly shorter than 11^th^.

Prothorax much longer than wide though propleura distinctly tumid, widest at about 1/3 from base; front much narrower than base, PAW/PBW=0.8; pronotum narrower than head, PW/HW=0.8–0.9 (mean 0.9), and much narrower than prothorax, PW/PTW=0.8; lateral borders almost parallel-sided at apical and basal parts, gently and slightly expanded at about 1/3 from base, slightly sinuate before hind angles; front angles right, hind angles obtuse (Fig. 32); two pairs of latero-marginal setae present, at about 2/5 from base and a little before hind angles respectively.

Elytra moderately elongate (Fig. 7), much longer than wide, EL/EW=2.0; almost twice as wide as prothorax, EW/PTW=1.9–2.0 (mean 2.0); widest at about apical 2/5, lateral sides distinctly narrowed towards base (Fig. 32); striae well defined and punctate, two (middle and preapical) dorsal pores present on 3^rd^ stria, at about 3/7 and apical 1/7 respectively; chaetotaxy of marginal umbilicate pores similar in *Dongodytes deharvengi*.

Male genitalia (Figs 39–40): Median lobe of aedeagus similar in *Dongodytes deharvengi*, but slightly shorter; copulatory piece indistinct in inner sac; in dorsal aspect the apical lobe broader apically, with sides slightly wider; right and left parameres each with four long setae apically.

#### Remarks.

Dongodytes (Dongodytodes) jinzhuensis sp. n. differs from other congeners by its extremely dilated head and narrowly elytral sides near base.

#### Etymology.

This new species is named after its type locality.

#### Material examined.

Holotype: male, Guangxi: Du’an: Gaoling: cave Jinzhu Dong II, 24°06.514N, 108°04.695E, 218 m, 2013-V-03, leg. Mingyi Tian, Weixin Liu, Feifei Sun & Haomin Yin, in SCAU; Paratypes: 1 males, 7 females, ibid; 3 males, 1 female, cave Jinzhu Dong I, 24°06.547 N, 108°04.785 E, 190 m, same date and collectors; all are in SCAU except one male paratype in MNHN.

#### Distribution.

China (Guangxi). Known only from caves Jinzhu Dong I and Jinzhu Dong II in Gaoling (Figs 1d and 73g).

**Figures 61–65.**
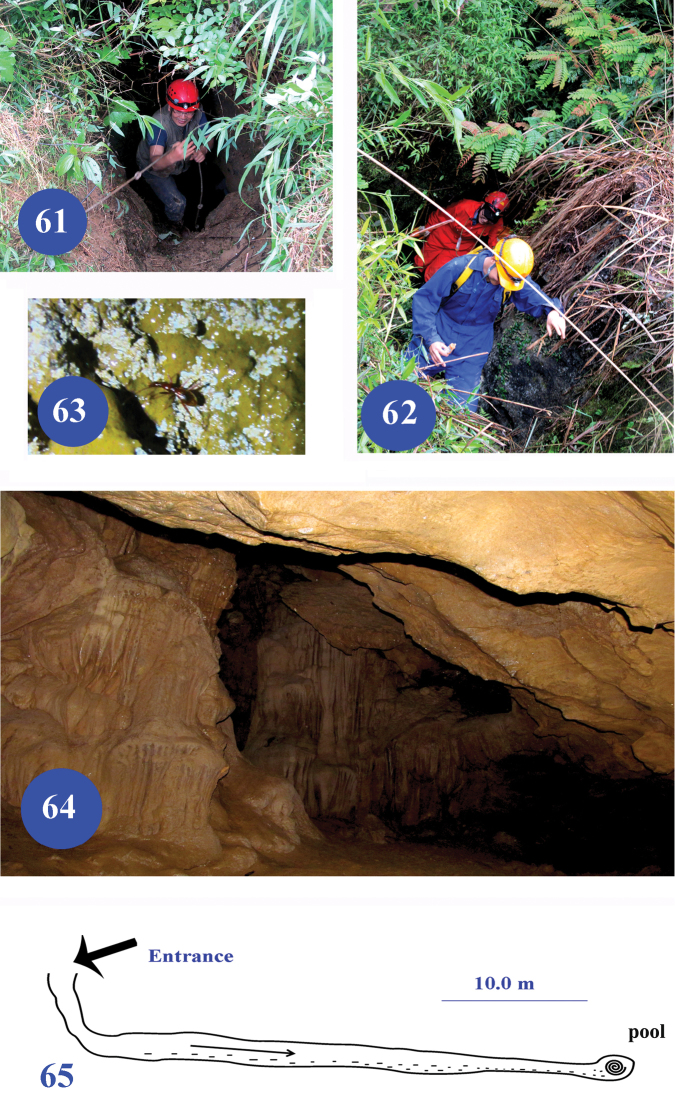
Jinzhu Dong I and II, type localities of Dongodytes (Dongodytodes) jinzhuensis sp. n. **61** entrance of Jinzhu Dong I **62** entrance of Jinzhu Dong II **63** wandering beetle on wall in Jinzhu Dong II **64** a room in Jinzhu Dong II, to show where the beetles were collected **65** a sketch of Jinzhu Dong I.

The entrance of Jinzhu Dong I (Figs 61, 65) is very narrow and almost vertical. The passage is about half to three metres wide, eight to twelve metres high, and about 40 m long, ending with a pool in which cave fishes are living. The trechine beetles were collected in a section at about 10 to 25 m from the entrance.

Jinzhu Dong II (Figs 62, 64) is about 200 m far from Jinzhu Dong I. The entrance is much bigger, having easier access than Jinzhu Dong I. The structure of this cave is complicated, which is composed of several big halls and many passages. The length is still unknown. The beetles were collected in wet areas from 20 to 40 m far from the entrance (Fig. 63).

Other animals living in above caves are crickets, millipedes, scutigers, isopods, snails and fishes.

### Dongodytes (Dongodytodes) inexpectatus sp. n.

Taxon classificationAnimaliaColeopteraCarabidae

http://zoobank.org/0A3CF02B-50DA-4543-9FC9-0CE55A1ACAA6

Figs 1e , 12 , 22 , 41 –42 , 66 –69 , 73h

#### Description.

Length: 6.2–7.0 (mean 6.6 mm); width: 1.6–2.0 mm (mean 1.83 mm). Habitus as in Fig. 12.

Colour: Light dark brown, palps pale.

Macrosculpture: Head and pronotum smooth, elytra vaguely punctate, body moderately shiny and wholly pubescent.

Microsculpture: Densely and more or less transversely striate on head and pronotum, faint on elytra.

Head rather elongate (Fig. 22), less stout than in *Dongodytes jinzhuensis* sp. n., distinctly longer than wide, HL/HW=2.7–2.9 (mean 2.8); widest at about middle from labrum, head excluding mandibles distinctly longer than pronotum (1.4–1.9 times, mean 1.6); head (including mandibles) plus prothorax as long as elytra; genae distinctly expanded posteriorly though less so than in *Dongodytes jinzhuensis* sp. n., collar-shaped neck distinct, head about 2.3 times wider than collar-shaped neck, suddenly constricted before collar-shaped neck; almost parallel-sided in median part; two pairs of supraorbital setae present, anterior ones slightly longer than the posterior which are distinctly longer than pubescent setae and easily recognized; clypeus 10-setose; a pair of suborbital setae present, close to neck constriction; labial suture well developed; mentum quadrisetose, setae at base and median parts respectively, basal pits distinct, mental tooth simple; submentum octosetose; ligula 12-setose apically; palps slender, 4^th^ maxillary palpomere slightly longer than 3^rd^, 2^nd^ labial palpomere much longer than 3^rd^, bisetose on inner margin; antennae extending at about apical 1/6 of elytra, 1^st^ antennomere distinctly longer than 2^nd^, which is the shortest, 3^rd^ the longest, over twice as long as 2^nd^, gradually shortened from 4^th^ until 10^th^, 11^th^ as long as 8^th^ and distinctly longer than 10^th^.

Prothorax much longer than wide though propleura distinctly tumid, widest at about 1/3 from base; pronotum narrower than head, PW/HW=0.9, and than prothorax, PW/PTW=0.7–0.8 (mean 0.8); front narrower than base, PAW/PBW=0.7–0.8 (mean 0.8); lateral borders almost parallel-sided in frontal half, then feebly expanded at about basal 1/3, distinctly sinuate before hind angles; front angles right, hind angles broadly obtuse; only basal pair of latero-marginal setae present, close to hind angles.

Elytra moderately elongate, much wider than prothorax, EW/PTW=1.5–2.0 (mean 1.8); much longer than wide, EL/EW=1.8–2.0 (mean 1.9); widest at about apical 1/3, lateral sides near base not well marked, and nearly straight; striae well marked, only preapical dorsal pore present, at about apical 1/6 of elytra; chaetotaxy similar in *Dongodytes deharvengi*.

Male genitalia (Figs 41–42): Median lobe of aedeagus much shorter than in *Dongodytes deharvengi*, widely and strongly arcuate, sagittal aileron moderately sized but very distinct, apex slender in profile; inner sac armed with a long and rather narrower copulatory piece about 2/5 as long as the median lobe; in dorsal view, apical lobe moderately in width, roundly truncate at apex, not parallel-sided at subapical part; right paramere thin at apex, with two long apical setae, left paramere stout, triangular, with four long setae at subapex and apex.

#### Etymology.

The name of this new species indicates that it was really a surprise for us to collect the type material in a cave which is very close to caves Jinzhu Dong I and Jinzhu Dong II (about 2 km only), the type localities of *Dongodytes jinzhuensis* sp. n.

#### Remarks.

*Dongodytes inexpectatus* sp. n. is similar to *Dongodytes brevipenis* sp. n., but it is easily distinguished from the latter by its stouter head and pronotum, and lateral sides of elytra distinctly sinuate against 1^st^ and 2^nd^ umbilicate pores (indistinctly sinuate in *Dongodytes brevipenis*). Compared to *Dongodytes jinzhuensis* sp. n., *Dongodytes inexpectatus* sp. n. is much slender and smaller, head much less expanded, clypeus 10-setose (sexsetose in *Dongodytes jinzhuensis*), and only posterior pair of latero-marginal setae present on pronotum (two pairs present in *Dongodytes jinzhuensis*).

#### Material examined.

Holotype: male, Guangxi: Du’an: Gaoling: Nongchi: cave Nongguangshang Dong II, 24°05.4263N, 108°04.5726E, 175 m, 2013-XII-27, leg. Mingyi Tian, Weixin Liu, Haomin Yin & Xiaozhu Luo, in SCAU; Paratypes: 2 females, ibid, in SCAU.

#### Distribution.

Guangxi (Du’an). Known only from the limestone cave called Nongguanshang Dong II in Gaoling (Figs 1e and 73h).

**Figures 66–69.**
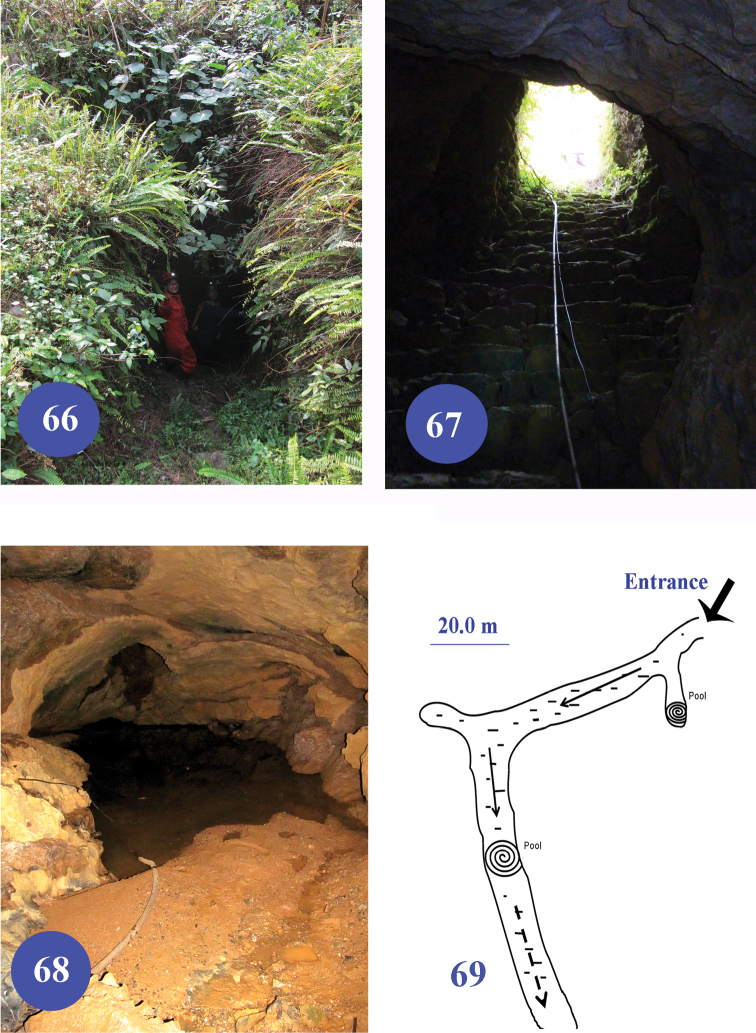
Nongguanshang Dong, type locality of Dongodytes (Dongodytodes) inexpectatus sp. n. **66–67** entrance of the cave **68** a pool at the end of the passage **69** a sketch of the cave.

The entrance of Nongguanshang Dong II (Figs 66–69) is in a corn field. Along the artificial steps it is easy to entre. It is a small cave, with a pool at about 30 m from the entrance which is water source of the local people. On the right of the pool, there is a gallery partially covered by water. The length of Nongguanshang Dong II is unknown and we surveyed up to about 100 m. The beetles were discovered on the ground and the wall in a small area on the gallery’s right side. Other cave animals found in the cave are crickets, spiders, isopods, shrimps, millipedes and snails.

### Dongodytes (Dongodytodes) yaophilus sp. n.

Taxon classificationAnimaliaColeopteraCarabidae

http://zoobank.org/6B5CB04C-AB89-4B4E-A81A-6345FD974265

Figs 1a , 15 , 24 , 45 –46 , 70 –73d

#### Description.

Length: 7.7–7.9 mm (mean 7.76 mm); width: 1.8–1.9 mm (mean 1.83 mm). Habitus as in Figs 15 and 72.

Colour: Less depigmented than other species of *Dongodytodes*, yellowish to dark reddish brown, palps pale.

Macrosculpture: Head and pronotum smooth, elytra vaguely punctate, body moderately shiny and wholly pubescent except mesostenum which is glabrous.

Microsculpture: Engraved meshes densely striate, more transverse on head and pronotum, and irregularly on elytra.

Head (Fig. 24) elongate, much longer than wide, HL/HW=3.0–3.1 (mean 3.1); widest at about middle including mandibles, excluding mandibles slightly longer than pronotum; genae moderately expanded posteriad, then suddenly constricted before the collar-shaped neck which is about 2/5 as wide as head; clypeus sexsetose; two pairs of supraorbital setae present, anterior ones far from each other and the posterior close to each other; a pair of suborbital setae present, mentum and submentum completely fused, mentum with two pairs of setae, at base and median part respectively, a pair of mental pits present; mental tooth bluntly bifid at apex; submentum 10-setose; 2^nd^ labial palpomere bisetose on inner margin; ligula multi-setose apically; antennae comparatively long, extending at about apical 1/8 of elytra; 1^st^ antennomere much more dilate than others, slightly longer than 2^nd^ which is shortest, 2^nd^ about half as long as 3^rd^ which is the longest, then gradually shortened towards to 10^th^ which is as long as 1^st^, and shorter than 11^th^.

Prothorax long, propleura less tumid than other species of the subgenus, PW/PTW=0.8–0.9 (mean 0.8); widest at about 2/7 from base, front distinctly narrower than base, PAW/PBW=0.7–0.8 (mean 0.7); pronotum slightly narrower than head, PW/HW=0.9; two pairs of latero-marginal setae present, at about 4/7 from base and a little before hind angles respectively, lateral sides distinctly sinuate just before hind angles; front angles right, hind angles obtuse.

Elytra more elongate and a little more convex than other species of *Dongodytodes*, as long as head (including mandibles) and pronotum combined; almost twice as long as wide, EL/EW=2.1; much wider than prothorax, EW/PTW=1.7–2.1 (mean 1.9); base rather slender, lateral sides near base almost straight; widest at about apical 4/7 of elytra; striae punctate, more or less traceable, two (middle and preapical) dorsal pores present on 3^rd^ stria, at about 3/5 and 1/6 respectively; chaetotaxy and other characters as in *Dongodytes deharvengi*.

Male genitalia (Figs 45–46): Median lobe of aedeagus long and slender, similar in *Dongodytes deharvengi*, but apical part more straight, and blunt at apex; inner sac armed with a large and long copulatory piece which covered with scale structures on surface, about 2/5 as long as the median lobe; in dorsal aspect the apical lobe broader and sides distinctly sinuate at subapical part; right and left parameres with three and four long setae at apex respectively.

#### Remarks.

By its long and large body, together with more elongate genae and longer antennae, *Dongodytes yaophilus* sp. n. is easily distinguished from other members of *Dongodytodes*. It is probably close to *Dongodytes deharvengi* because both of them have similar aedeagal structure and elytral chaetotaxal pattern.

#### Etymology.

“Yao” is a short name for the minority Yao people who are living in the mostly karstic mountainous areas in several provinces or regions of southern China (Guangxi, Hunan, Guangdong and Guizhou). Both Dahua and Du’an are Yao Autonomous Counties. The name indicates that this new species lives in the same region as Yao people.

#### Material examined.

Holotype: male, Guangxi: Dahua: Qibainong: cave Qiaoxu Dong, 24°04.370N, 107°40.140E, 535 m, 2013-VI-22, leg. Mingyi Tian, Wei Lin, Haomin Yin & Sunbin Huang; Paratypes: 3 females, ibid, all in SCAU.

#### Distribution.

Guangxi (Dahua) (Figs 1a and 73d). Known only from the type locality, cave Qiaoxu Dong.

**Figures 70–72.**
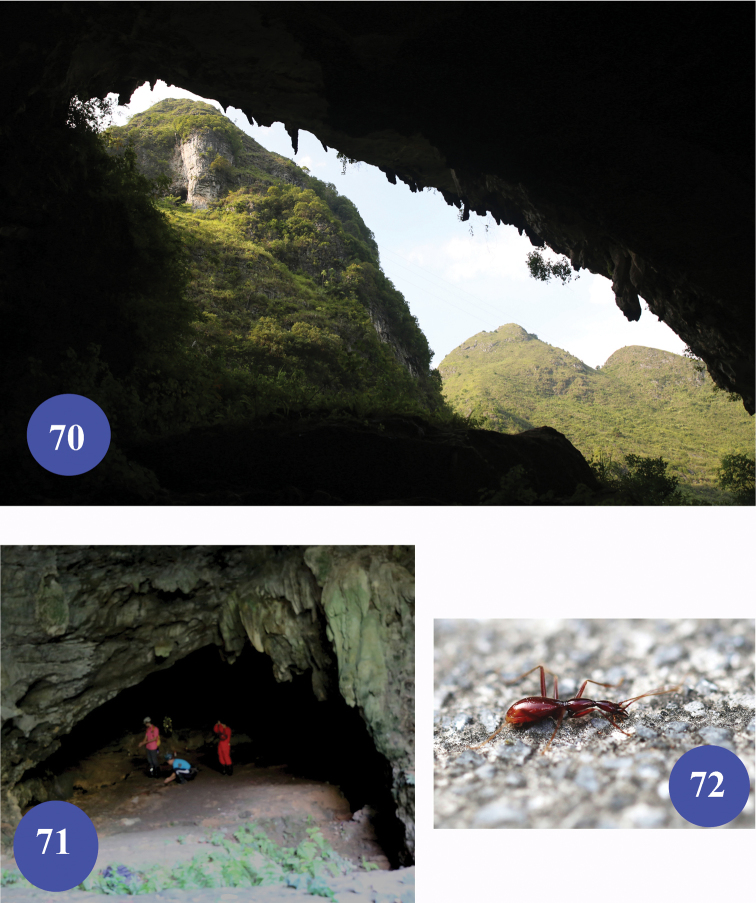
Qiaoxu Dong, type locality of Dongodytes (Dongodytodes) yaophilus sp. n. **70–71** entrance of the cave **72** a wandering beetle on ground.

Qiaoxu Dong (Figs 70–71) is located at about 250 m from village Qiaoxu in the west, along the main road from Qibainong to Dahua. It is a large cave, 420 m long, 15 to 140 m wide and six to 30 m high, having three large halls. The largest hall is about 336, 000 m^2^ in area, one of the largest in Guangxi. It is a beautiful cave within Qibainong National Geopark in Dahua County. The beetles were collected in the areas of 70 to 100 m from the entrance. Other cave animals in Qiaoxu Dong are crickets, spiders, millipedes, isopods and snails.

**Figure 73.**
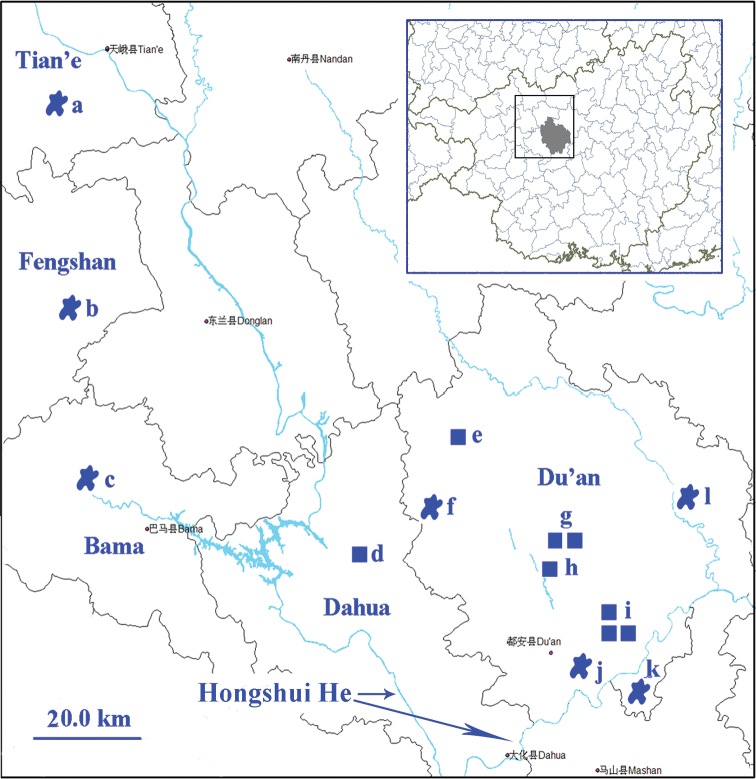
Distribution of the genus *Dongodytes*. **a** Dongodytes (s. str.) giraffa Uéno **b** Dongodytes (s. str.) grandis Uéno **c** Dongodytes (s. str.) fowleri Deuve **d** Dongodytes (Dongodytodes) yaophilus sp. n. **e** Dongodytes (Dongodytodes) deharvengi Tian **f** Dongodytes (s. str.) troglodytes sp. n. **g** Dongodytes (Dongodytodes) jinzhuensis sp. n. **h** Dongodytes (Dongodytodes) inexpectatus sp. n. **i** Dongodytes (Dongodytodes) brevipenis sp. n. **j** Dongodytes (s. str.) baxian Tian **k** Dongodytes (s. str.) lani sp. n. **l** Dongodytes (s. str.) elongatus sp. n. Star: species of *Dongodytes* (s. str.); square: species of *Dongodytodes*.

## Supplementary Material

XML Treatment for Dongodytes

XML Treatment for Dongodytes (s. str.)

XML Treatment for Dongodytes (s. str.) baxian

XML Treatment for Dongodytes (s. str.) elongatus

XML Treatment for Dongodytes (s. str.) troglodytes

XML Treatment for Dongodytes (s. str.) lani

XML Treatment for Dongodytodes

XML Treatment for Dongodytes (Dongodytodes) deharvengi

XML Treatment for Dongodytes (Dongodytodes) brevipenis

XML Treatment for Dongodytes (Dongodytodes) jinzhuensis

XML Treatment for Dongodytes (Dongodytodes) inexpectatus

XML Treatment for Dongodytes (Dongodytodes) yaophilus
